# Extracts of*Hylotelephiumerythrostictum* (miq.) H. Ohba ameliorate intestinal injury by scavenging ROS and inhibiting multiple signaling pathways in *Drosophila*

**DOI:** 10.1186/s12906-024-04686-w

**Published:** 2024-11-14

**Authors:** Hyonil Kim, Xinyu Yi, Hongmei Xue, Guanhua Yue, Jiahua Zhu, Tongju Eh, Sihong Wang, Li Hua Jin

**Affiliations:** 1 College of Life Science, Northeast Forestry University, Harbin, Heilongjiang Province China; 2 College of LifeScience, Kim Il Sung University, Pyongyang, Democratic People’s Republic of Korea; 3 Women and Children’s Hospital, Peking University People’s Hospital, Qingdao University, Qingdao, China; 4 Analysis and Test Center, Yanbian University, Yanji, 133002 Jilin Province PR China; 5 Department of Basic Medical, Shenyang Medical College, Shenyang, China

**Keywords:** *Hylotelephiumerythrostictum*, Inflammatory bowel disease, *Drosophila*, Intestinal homeostasis, Intestinal stem cell, Pathway

## Abstract

**Background:**

The intestinal epithelial barrier is the first line of defense against pathogens and noxious substances entering the body from the outside world. Through proliferation and differentiation, intestinal stem cells play vital roles in tissue regeneration, repair, and the maintenance of intestinal homeostasis. Inflammatory bowel disease (IBD) is caused by the disruption of intestinal homeostasis through the invasion of toxic compounds and pathogenic microorganisms. *Hylotelephium erythrostictum* (Miq.) H. Ohba (*H. erythrostictum*) is a plant with diverse pharmacological properties, including antioxidant, anti-inflammatory, antidiabetic, and antirheumatic properties. However, the roles of *H. erythrostictum* and its bioactive compounds in the treatment of intestinal injury are unknown.

**Methods:**

We examined the protective effects of *H. erythrostictum* water extract (HEWE) and *H. erythrostictum* butanol extract (HEBE) on *Drosophila* intestinal injury caused by dextran sodium sulfate (DSS) or *Erwinia carotovoracarotovora 15* (*Ecc15*).

**Results:**

Our findings demonstrated that both HEWE and HEBE significantly prolonged the lifespan of flies fed toxic compounds, reduced cell mortality, and maintained intestinal integrity and gut acid‒base homeostasis. Furthermore, both HEWE and HEBE eliminated DSS-induced ROS accumulation, alleviated the increases in antimicrobial peptides(AMPs) and intestinal lipid droplets caused by *Ecc15* infection, and prevented excessive ISC proliferation and differentiation by inhibiting the JNK, EGFR, and JAK/STAT pathways. In addition, they reversed the significant changes in the proportions of the gut microbiota induced by DSS. The bioactive compounds contained in *H. erythrostictum* extracts have sufficient potential for use as natural therapeutic agents for the treatment of IBD in humans.

**Conclusion:**

Our results suggest that HEWE and HEBE are highly effective in reducing intestinal inflammation and thus have the potential to be viable therapeutic agents for the treatment of gut inflammation.

**Clinical trial number:**

Not applicable.

**Supplementary Information:**

The online version contains supplementary material available at 10.1186/s12906-024-04686-w.

## Introduction

Inflammatory bowel disease (IBD) is a chronic disease that causes intestinal injury and disturbance and affects the gastrointestinal tract, with Crohn’s disease (CD) and ulcerative colitis (UC) being the most prevalent forms [1]. As IBD is difficult to treat completely and many medications reduce only symptoms, there is a need for more effective therapeutic medicines for IBD [2]. *Drosophila melanogaster* has become an outstanding model for studying disease mechanisms and drug screening in recent years [3]. The gastrointestinal tract of flies is composed of self-regenerating digestive and absorbent tissues and has similar properties to their mammalian counterparts, including the stomach, small intestine, and colon [4].Compared to those of the human small intestine, the posterior midgut possesses the highest metabolic activity and immune response [5]. When a fly consumes toxic chemicals or microorganisms, intestinal homeostasis is disrupted, leading to the development of IBD. As a result, the *Drosophila* intestine has become an ideal subject for IBD research.

The*Drosophila* intestinal epithelium contains four distinct cell types: intestinal stem cells (ISCs), enteroblasts (EBs), enterocytes (ECs), and enteroendocrine cells (EEs). Through self-renewal and differentiation processes, ISCs play a crucial role in maintaining intestinal homeostasis [5, 6]. *Drosophila* intestinal epithelial homeostasis is maintained by a number of signaling pathways. The Notch signaling pathway initiates ISC differentiation, identifies EB terminal cell fates, and possibly inhibits erroneous ISC proliferation, thereby decreasing ISC turnover [5]. Moreover, EGFR signaling plays a significant role in regulating ISC proliferation [7]. The JAK/STAT and c-Jun N-terminal kinase (JNK) signaling pathways are involved in inflammation and infection and promote ISC proliferation and differentiation. The microbiota also modulates the activity of ISCs by causing cell death and replacement, as well as modifying the physiology of the gut and the body as a whole [8].

Recent research efforts have focused on the development of new and efficacious natural medications for the treatment of IBD. *H. erythrostictum* is a perennial herbaceous plant with fleshy leaves that belongs to the *Crassulaceae* family. *H. erythrostictum*, commonly known as the garden stonecrop, has been widely applied in urban greening and ecological gardening in China, due to its remarkable ornamental value and strong stress resistance [9, 10]. *H. erythrostictum* is widely recognized as a multifunctional plant due to its exceptional pharmacological and ornamental properties. *H. erythrostictum* has also been used as a traditional Chinese medicine, and according to the Chinese encyclopedia dictionary “Cihai”, the whole plant is employed as a therapeutic agent for the treatment of rheumatism and diabetes [11]. Extracts from the aerial portions of *H. erythrostictum* were found to have potent antioxidant and anti-inflammatory properties [10]. In addition, the chemical composition and antibacterial activity of the ethyl acetate extract were revealed [12]. Previous research on *H. erythrostictum* has focused on drought stress [13], salt stress [14], chloroplast genome completion [9], and α-glucosidase inhibition [11]. However, the protective effects of *H. erythrostictum* extracts and their bioactive compounds against intestinal injury have not been determined.

Using *Drosophila melanogaster* as a model organism, this study revealed the protective function and regulatory mechanism of *H. erythrostictum* water extract (HEWE) and butanol extract (HEBE) against the disruption of intestinal homeostasis caused by the ingestion of dextran sodium sulfate (DSS) or *Erwinia carotovoracarotovora 15* (*Ecc15*) infection. In addition, the functional compounds of these extracts were analyzed. Supplementation with HEWE or HEBE significantly increased adult fly survival and restored ROS levels, acid‒base homeostasis, and midgut lipid droplet accumulation induced by DSS or *Ecc15*, according to our findings. Furthermore, both HEWE and HEBE inhibited ISC proliferation and differentiation by inhibiting the activation of the JNK, EGFR, and JAK/STAT signaling pathways. Additionally, both HEWE and HEBE restored the equilibrium of the disturbed intestinal microflora and preserved intestinal homeostasis. Moreover, several effective compounds for treating inflammatory bowel disease were identified in both HEWE and HEBE. In conclusion, our results demonstrated that *H. erythrostictum* extracts possess a distinct mechanism of protection against intestinal damage and have the potential to serve as a novel, effective treatment for gut inflammation.

## Materials and methods

### *Hylotelephium erythrotictum* water extract (HEWE) and butanol extract (HEBE)

*Hylotelephiumerythrostictum* (Miq.) H. Ohba (Crassulaceae) was collected in September 2013 on the campus of Yanbian University in Yanji City, identified by Professor Huizi Lv (College of Pharmacy, Yanbian University). A voucher specimen (number: YB-HE-1309) was deposited at the Pharmacognosy Laboratory, College of Pharmacy, Yanbian University. The fresh medicinal plant stems were dried in the shade until their true quality remained unchanged and were then crushed in a grinder. All filtration operations were carried out in a Buchner funnel under vacuum. A total of 185 g of desiccated *H. erythrostictum* stems was macerated with 70% EtOH (200 ml×5) for 3 h (each time) under reflux in a 1 L three-necked round-bottomed flask in an oil bath with a drug‒solvent ratio (DSR) = 1:1. Under vacuum, the combined extracts were concentrated to produce a crude extract. After being suspended in water (20 ml), the crude extract was successively partitioned with petroleum ether (20 ml×3, resulting in a drug-to-extract ratio (DER) = 1:10), dichloromethane (20 ml×3, resulting in a drug-to-extract ratio (DER) = 1:18), ethyl acetate (20 ml×3, resulting in a drug-to-extract ratio (DER) = 1:19), and n-butanol (20 ml×3, resulting in a drug-to-extract ratio (DER) = 1:5) in a 500 ml separating funnel at normal pressure and temperature. All the extracts were dried under vacuum drying mode. Accordingly, 1.19 g of dry water-insoluble extract, 1.76 g of dry petroleum ether extract, 1.34 g of dry dichloromethane extract, 1.25 g of dry ethyl acetate extract, 4.10 g of dry n-butanol extract, and 44.67 g of dry water-soluble extract were obtained. For experimental repeatability, the obtained water extract (10 mg) and n-butanol extract (10 mg) were dissolved in deuterated methanol, after which the nuclear magnetic resonance (NMR) hydrogen and carbon spectra were measured on a Bruker AV500 nuclear magnetic resonance spectrometer for fingerprint authentication and standardization.

### Drosophila stocks

All flies used in this study were as follows: the *esg-Gal4 UAS-GFP/CyO* line was obtained from Rongwen Xi [15]. The *Delta-lacZ* line was a gift from Bruno Lemaitre [16]. Jose Carlos Pastor-Pareja donated the *upd3-Gal4 UAS-GFP/CyO* line [17]. The Bloomington Stock Center was the source for the *w*^*1118*^, *Vn-lacZ*, *puc-lacZ*, *UAS-hep*^*WT*^*/CyO*, *GstD1-GFP*, *UAS-EGFR*^*CA*^, *UAS-Cat RNAi*, *UAS-Sod2 RNAi*, and *UAS-hop*^*CA*^ lines. The Tsinghua Fly Center was the source for the *Su(H)GBE-lacZ*, *esg*^*ts*^*-Gal4 UAS-GFP*, *10XSTAT-GFP*, and *Dipt-GFP* lines. The *Def-GFP* line was generated by Jean-Luc Imler [18]. The *Drs-GFP* line was provided by Zongzhao Zhai as a gift [19]. All flies were maintained on a standard diet of cornmeal and yeast at 25 °C and 60% humidity with a 12-hour light cycle.

### Survival experiments

Three- to five-day-old adult *w*^*1118*^ flies (thirty per group, half female and half male) were used as experimental animals for survival experiments. After starving for 2 h, the flies were transferred to plastic vials containing 5 layers of filter paper soaked in 5% sucrose medium (w/v), 5% sucrose medium containing 4% DSS (w/v; MP Biomedicals, LLC, Santa Ana, CA), 5% sucrose medium containing 0.6% SDS (w/v; Sigma‒Aldrich), and DSS or SDS medium supplemented with HEWE (4, 6, and 8 mg/ml) or HEBE (1, 2, and 4 mg/ml). The filter papers were changed daily at the same time, and survival rates were recorded daily. Three independent experimental repeats were performed.

### Immunostaining

The intestines of 15 to 20 female flies per group were dissected in cool PBS and then fixed for 30 min at room temperature in 4% PFA (paraformaldehyde). After fixation, the samples were flushed four times (for 10 min each) in blocking buffer (PBST containing 5% goat serum) and blocked for 30 min. Then, the samples were incubated overnight at 4 °C with primary antibodies in the same buffer. After washing with PBST four times for 10 min each, the intestines were incubated for two hours at room temperature with secondary fluorophore-conjugated antibodies in PBST. The samples were cleaned a total of 10 times for 5 min in PBST, stained with Hoechst (1:500 in PBST), and then mounted in glycerol at a concentration of 70%. A Carl Zeiss Axioscope A1 microscope was used to acquire the images. The primary antibodies used were as follows: rabbit anti-PH3 (1:200, Millipore), mouse anti-β-gal (1:200, Promega), rabbit anti-GFP (1:200, Thermo Fisher Scientific), mouse anti-Prospero (1:200, DSHB), mouse anti-armadillo (Arm) (1:30, DSHB), rabbit anti-p-JNK (1:200, Promega), and rabbit anti-p-Erk (1:50, Cell Signaling Technology). At a ratio of 1:200, the secondary antibodies used were Alexa Fluor 488- and Alexa Fluor 568-conjugated antibodies (Thermo Fisher Scientific). Three independent experimental repeats were performed.

### BODIPY staining

Adult intestines were dissected, fixed in 4% paraformaldehyde for 30 min at room temperature, and washed four times for 5 min with PBST (0.1% Triton X-100 in PBS). The samples were stained with 2 µg/ml BODIPY and incubated for 1 h. The excess stain was then removed, and each sample was rinsed three times for 5 min in PBST. Next, the samples were incubated with 2 µg/ml Hoechst for 10 min before being mounted on glass transparencies with 70% glycerol. Three independent experimental repeats were performed.

### Monitoring gut acidification

To determine the acidity of the *Drosophila* intestine, 100 ml of 2% BPB (bromophenol blue sodium; Sigma, B5525) was deposited on the surface of the food, and a pipette tip was used to ensure that the BPB solution was absorbed. After 12 h, the intestines were dissected and photographed in ice-cold PBS. Three independent experimental repeats were performed.

### Quantitative real-time PCR for AMP expression

TRIzol (Thermo Fisher Scientific) was used to extract total RNA from 30 guts per group, and cDNA was synthesized using M-MLV Reverse Transcriptase RNase H Minus Point Mutant (Promega). Quantitative real-time PCR was performed on a LightCycler 480 II (Roche) using ROX FastStart Universal SYBR Green Master Mix reagents. The following primers were used in the experiments: *rp49*: F-AGTCGGATCGATATGCTAAGCTGT and R-TAACCGATGTTGGGCATCAGATACT; *Drs* (*drosomycin*): F-CTTGTTCGCCCTCTTCGCTGTC and R-AGCACTTCAGACTGGGGCTGCA; *Dpt* (*diptericin*): F-ATGCAGTTCACCATTGCCGTC and R-TCCAGCTCGGTTCTGAGTTG; *Def* (*defencin*): F-CGCTTTTGCTCTGCTTGCTTGC and R-TAGGTCGCATGTGGCTCGCTTC. As an internal reference, relative AMP expression was normalized to that of the *rp49* gene. Five independent experimental repeats were performed.

### Smurf assay

The Smurf assay, which was used to assess the intestinal barrier integrity of *Drosophila*, was performed using 2.5% (w/v) blue dye (FD&C Blue #1) in accordance with a previously described method [20]. After 5 days of feeding, the female flies were transferred to medium containing blue dye. The ‘Smurf’ phenotype was observed after 9 h if the fly’s entire body was determined to be blue. Three independent experimental repeats were performed.

### Bacterial cultures and oral infection

*Ecc15* was grown in LB medium at 29 °C for 12 h. Female flies aged 3 to 5 days were starved for 2 h at 25 °C before being transferred to receptacles containing five pieces of filter paper. As a feeding medium, filter papers were saturated with 5% sucrose, and *Ecc15* bacteria (final OD600_nm_ = 200) were cultured in 5% sucrose.

### 7-Aminoactinomycin D (7-AAD), DHE, and CM-H2DCFDA staining

7-AAD staining was used to identify dead cells, while DHE and CM-H_2_DCFDA staining were used to measure intestinal ROS levels. The intestines of female adult flies were dissected in PBS and stained for 30 min at room temperature with 5 µM 7-AAD, 5 µM DHE, or 1 µM CM-H_2_DCFDA. The dissected intestines were then fixed in 4% PFA and stained with Hoechst for 10 min. Finally, the digestive tract was affixed with 70% glycerol. Three independent experimental repeats were performed.

### Gut microbiota analysis

Using 16 S rDNA analysis, the intestinal microbial composition of *Drosophila* was analyzed. Using the Fast DNA Spin Kit, intestinal microbial DNA was extracted, and the extracted genomic DNA was analyzed via 1% agarose gel electrophoresis. The sequencing results for 16 S rDNA were obtained from Shanghai Majorbio Bio-Pharm Technology Co., Ltd. A paired-end library was constructed using the NEXTFLEX Rapid DNASeq Kit in accordance with Illumina’s recommendations for genomic DNA library preparation. The amplicons were subsequently sequenced with a MiSeq Reagent Kit v3.

### Widely targeted metabolomics

*H. erythrostictum* extracts were subjected to metabolite profiling at Shanghai Hoogen Biotechnology Co., Ltd. (Shanghai, China) using a widely targeted metabolomic method (http://www.hoogen-bio.com/). The following conditions were used for ultracentrifugation (UCPLC) analysis. The column used was an Acquity UPLC HSS T3 (1.8 μm, 2.1 × 100 mm) column; mobile phase A was 0.1% formic acid in water, and mobile phase B was acetonitrile. A gradient of water/acetonitrile was applied, starting with a ratio of 98:2 V(A)/V(B) at 0 min, maintaining this ratio until 0.5 min, then transitioning to 50:50 V(A)/V(B) at 10 min, 5:95 V(A)/V(B) at 11.0 min, 5:95 V(A)/V(B) at 13.0 min, returning to 98:2 V(A)/V(B) at 13.1 min, and finally maintaining this ratio until 15.0 min. The flow rate was 0.40 ml/min, the temperature of the column was 40 °C, the temperature of the autosampler was 4 °C, and the injection volume was 2 µl. The UHPLC instrument was integrated with tandem mass spectrometry (MS/MS) (Applied SCIEX 6500 QTRAP + triple quadrupole with an IonDrive Turbo V ESI ion source). The following were the MS conditions: IonSpray Voltage was set to + 5500/-4500 V, Curtain Gas was set to 35 psi, Temperature was set to 400 °C, Ion Source Gas 1 was set to 60 psi, Ion Source Gas 2 was also set to 60 psi, and DP was set to ± 100 V. SCIEX Analyst Workstation Software (Version 1.6.3) was used to acquire and evaluate the MRM (multiple reaction monitoring) method’s data. Using MSconverter, the original MS data (wiff) files were converted to the TXT format. Using an in-house R program and database, peak detection and annotation were performed.

### Targets prediction of HEWE and HEBE

Compounds with relative percentages > 0.1% obtained as SMILES in PubChem were identified through widely targeted metabolomics. The SwissADME platform was subsequently used to predict the ADME properties of these compounds, with a focus on identifying compounds with high gastrointestinal absorption and excellent drug-like properties. Subsequently, the SwissTarget prediction platform was used to predict potential targets of the selected compounds. For prospective drug targets, only those with a probability greater than 0.1% were considered.

### IBD-related and hub-related objectives

To identify targets associated with inflammatory bowel disease (IBD), several databases, including OMIM, CTD, DisGeNet, GeneCards, and TTD [21–24] were used. Direct evidence of correlation was the criterion for selecting targets from CTD. In DisGeNet, targets with scores greater than 0.02 were obtained. The GeneCards tool was used to screen for targets with correlation coefficients greater than the median. To normalize the datasets, UniProt [25] was used. The resulting targets were visualized as Venn diagrams by jvenn [26].

### Statistical analysis

ImageJ was used for image analysis, GraphPad Prism 6.0 was used for graph generation and results analysis by Student’s t test. Normality is analyzed using the Kolmogorov-Smirnov test.All data were shown as means ± SEM.*P* values > 0.05 indicated no significance. *P* < 0.05 was considered to indicate statistical significance (*P < 0.05; **P < 0.01; ***P < 0.001; ****P < 0.0001).

## Results

### *H. erythrostictum*extracts enhance protection against toxic compounds

**Fig. 1:**
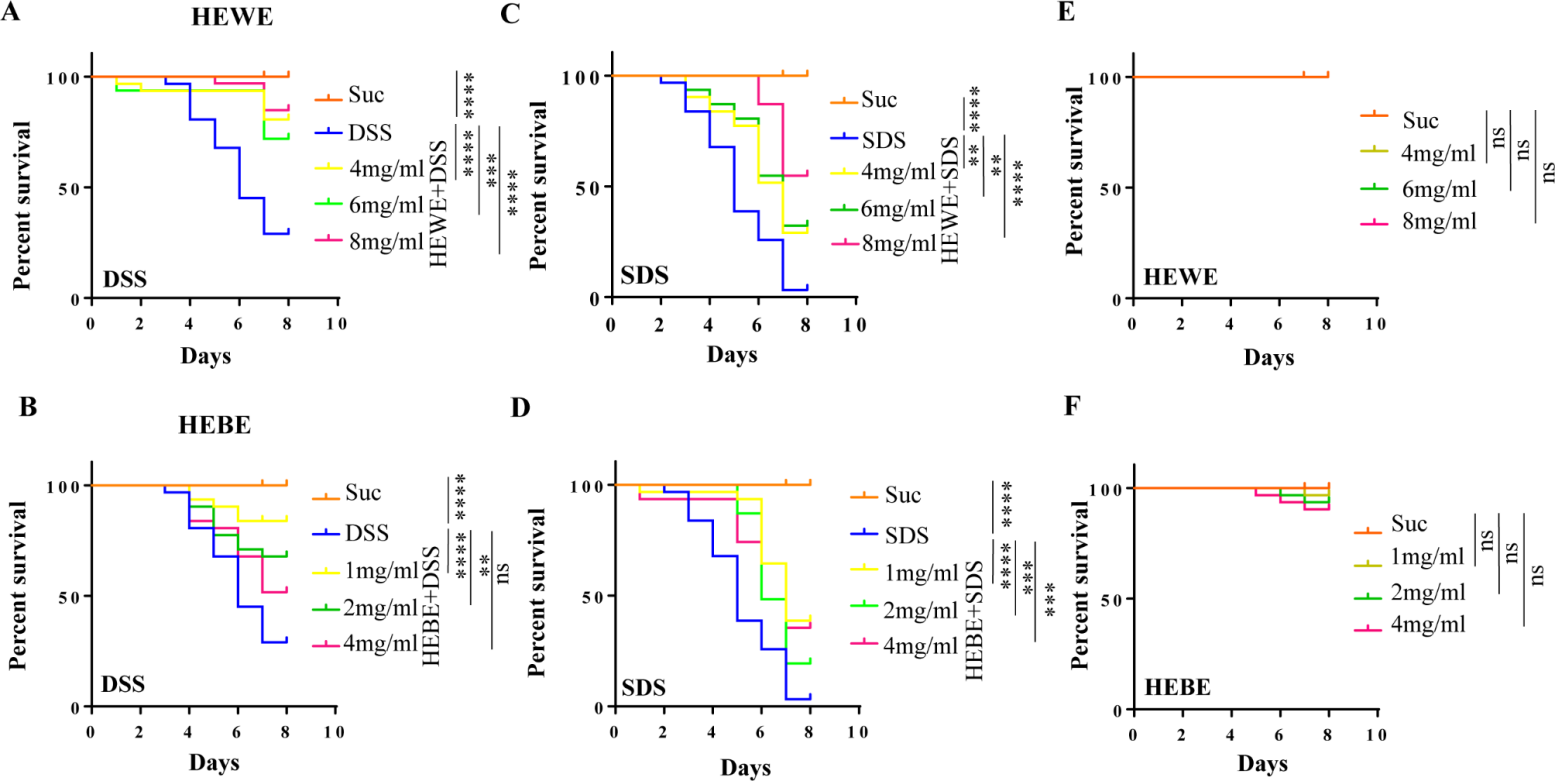
Both HEWE and HEBE enhance the survival of flies exposed to DSS or SDS. (**A**-**D**) Wild type *w*^*1118*^ flies were reared on various media. Suc, 5% sucrose medium; DSS, 5% sucrose containing 4% DSS; SDS, 5% sucrose containing 0.6% SDS; experimental group, DSS or SDS medium supplemented with HEWE (4, 6, and 8 mg/ml) or HEBE (1, 2, and 4 mg/ml), respectively. Supplementation with HEWE or HEBE at each concentration significantly increased the survival rate of flies exposed to toxic compounds. (**E**, **F**) Flies were reared in 5% sucrose medium supplemented with HEWE (**E**) or HEBE (**F**) without toxic compounds. Neither HEWE nor HEBE at any concentration was harmful to fruit flies. Differences in survival were analyzed using the log-rank test. ns*P* > 0.05,**P < 0.01,***P < 0.001,****P < 0.0001

To determine whether *H. erythrostictum* water extract (HEWE) and butanol extract (HEBE) have protective effects against toxic substances in the intestine, adult flies were fed DSS or SDS, respectively. These chemicals are toxic compounds that disrupt normal intestinal barrier function and cause intestinal inflammation. To determine the survival rate, adult flies were fed multiple concentrations of HEWE (4, 6, and 8 mg/ml) or HEBE (1, 2, and 4 mg/ml). After 7 days, the survival rates of the flies in the 4% DSS and 0.6% SDS groups substantially decreased by 71% and 96.8%, respectively (Fig. 1A-D). However, compared to those in the control groups, the survival rates of the experimental groups administered HEWE or HEBE were significantly greater, with 8 mg/ml HEWE and 1 mg/ml HEBE having the greatest protective effects. After exposure to 4% DSS, supplementation with 8 mg/ml HEWE or 1 mg/ml HEBE increased survival by 55.8% (*P <* 0.0001) and 54.2% (*P <* 0.0001), respectively (Fig. 1A, B). The survival rates increased substantially by 51.6% (*P <* 0.0001) and 35.5% (*P <* 0.0001), respectively, when 0.6% SDS was utilized as an inflammatory agent. (Fig. 1C, D). The toxicity of *H. erythrostictum* extracts to *Drosophila* was subsequently evaluated. As shown in Fig. 1E and F, the survival rates of the group fed HEWE or HEBE did not differ from the survival rate of the group fed sucrose alone, indicating that the *H. erythrostictum* extracts were not toxic to *Drosophila*. Overall, 8 mg/ml HEWE and 1 mg/ml HEBE significantly enhanced the survival rates of flies exposed to the inflammatory factors DSS and SDS. Next, we used only DSS to induce intestinal dysfunction.

### *H. erythrostictum* extracts protect against the degradation and dysfunction of intestinal epithelial cells caused by DSS

**Fig. 2:**
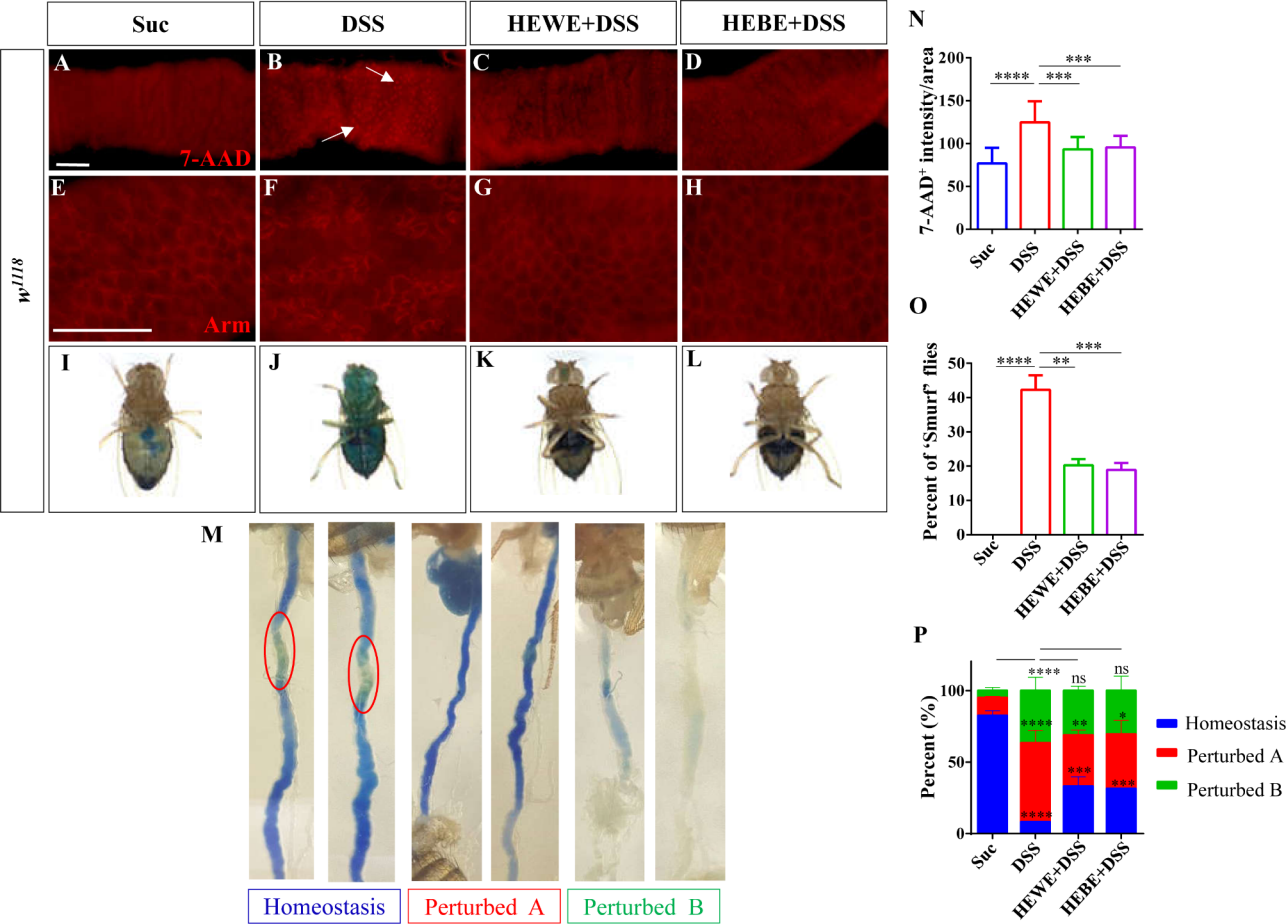
Both HEWE and HEBE can prevent DSS-induced intestinal epithelial cell death and dysfunction. (**A**-**D**) *w*^*1118*^ flies were exposed to 6% DSS for 72 h, and 7-AAD was used to detect dead cells. The addition of HEWE or HEBE significantly reduced DSS-induced cell death. (**E**-**H**) The cell membranes were stained with anti-Arm antibodies. Following the administration of DSS, the intestinal cell arrangement was disrupted; however, supplementation with HEWE or HEBE restored the aberrant cellular arrangement. (**I**-**L**) Images of ‘Smurf’ flies on different foods. ‘Smurf’ flies discharge blue dye from the intestine into other tissues. (**M**) Illustration of intestinal acid‒base homeostasis indicated by the bromophenol blue pH indicator. ‘Homeostasis’ indicates that the CCR is yellow, ‘Perturbed A’ indicates that the CCR is blue, and ‘Perturbed B’ indicates that the flies were not administered bromophenol blue and that the intestinal tract was not stained with a pH indicator. (**N**) Fluorescence intensity of 7-AAD as measured in A-D (*n* = 12–16). Three separate experimental repeats were performed. (**O**) Quantification of the percentage of Smurf flies in I-L. Sixty flies were included in each group. Three separate experimental repeats were performed. (**P**) The percentage of intestinal acid‒base homeostasis in M. Each group consisted of 60 flies. Three experimental repeats were performed. Quantification results represent mean ± SEM.ns *P* > 0.05, *P < 0.05, **P < 0.01,***P < 0.001,****P < 0.0001. Scale bars: 50 μm

As *H. erythrostictum* extracts improved *Drosophila* survival after ingestion of toxic compounds, we hypothesized that they may protect intestinal function. 7-AAD staining was used to evaluate DSS-induced intestinal epithelial cell death. The fluorescence intensity of 7-AAD was substantially greater in DSS-treated flies than in sucrose-fed flies (*P <* 0.0001, Fig. 2A, B, N). However, the fluorescence signal decreased by more than 76% after supplementation with *H. erythrostictum* extracts (HEWE:*P <* 0.001, HEBE: *P <* 0.001, Fig. 2C, D, N). In addition, adherens junctions regulate cell adhesion and cytoskeletal organization in a variety of cell types. Armadillo (Arm) is a crucial protein that binds to other proteins to form a cell‒cell junction complex [27]. Previous research has demonstrated that DSS stimulation disrupts and disorganizes the adhesion of intestinal epithelial cells [28]. To assess cell adhesion against intestinal damage, we utilized anti-Arm antibodies. In the sucrose-fed group, clear small progenitor cells and large ECs, which make up the majority of the intestinal epithelium, were observed, whereas in the DSS-treated group, cell boundaries were disorganized and cell types could not be distinguished (Fig. 2E, F). After supplementation with HEWE or HEBE, the cell adhesions resembled those of the sucrose-fed group in terms of clarity and orderliness (Fig. 2G-H).

Cell mortality and disordered cell arrangement may induce disruption of the epithelial barrier, thereby increasing intestinal permeability. Next, we determined the integrity of the intestinal epithelial barrier in a Smurf assay. In the Smurf experiment, a nonabsorbable blue food dye (FD&C Blue #1) was used, and flies whose entire bodies exhibited blue color after ingesting the dye were referred to as ‘Smurf’ flies [29]. In the DSS-fed group, 42% of flies exhibited the ‘Smurf’ phenotype (*P <* 0.0001, Fig. 2I, J, O). In contrast, supplementation with 8 mg/ml HEWE or 1 mg/ml HEBE significantly reduced the Smurf phenotype to 20% (*P <* 0.01) and 18% (*P <* 0.001), respectively (Fig. 2K, L, O).

Previous research has suggested that intestinal acid‒base homeostasis plays an essential role in metabolic regulation [30], and instability of intestinal epithelial cells results in a loss of acid‒base homeostasis [31]. The copper cell region (CCR) of fruit flies is located in the middle midgut and is composed of a group of copper cells implicated in gastric acid secretion [32]. Bromophenol blue (BPB) is a pH sensitive dye that transforms from yellow at pH 3.0 to blue at pH 4.6 or higher. Therefore, when *Drosophila* consumes BPB, the CCR under intestinal homeostasis appears yellow. We used BPB to determine whether DSS administration could disrupt the acid‒base homeostasis of CCR in the intestine. In the DSS-fed group, acid-base homeostasis was significantly reduced by 74.5% (*P <* 0.0001) compared to that in the sucrose-fed group; however, in the HEWE- and HEBE-supplemented groups, acid-base homeostasis was significantly improved by 25% (*P <* 0.001) and 23.4% (*P <* 0.001), respectively, compared to that in the DSS-fed group (Fig. 2M, P). These results suggested that *H. erythrostictum* extracts could protect against the disruption of intestinal morphology and function caused by DSS administration.

### *H. erythrostictum* extracts reduce excessive AMP levels and steatosis caused by *Ecc15*

**Fig. 3:**
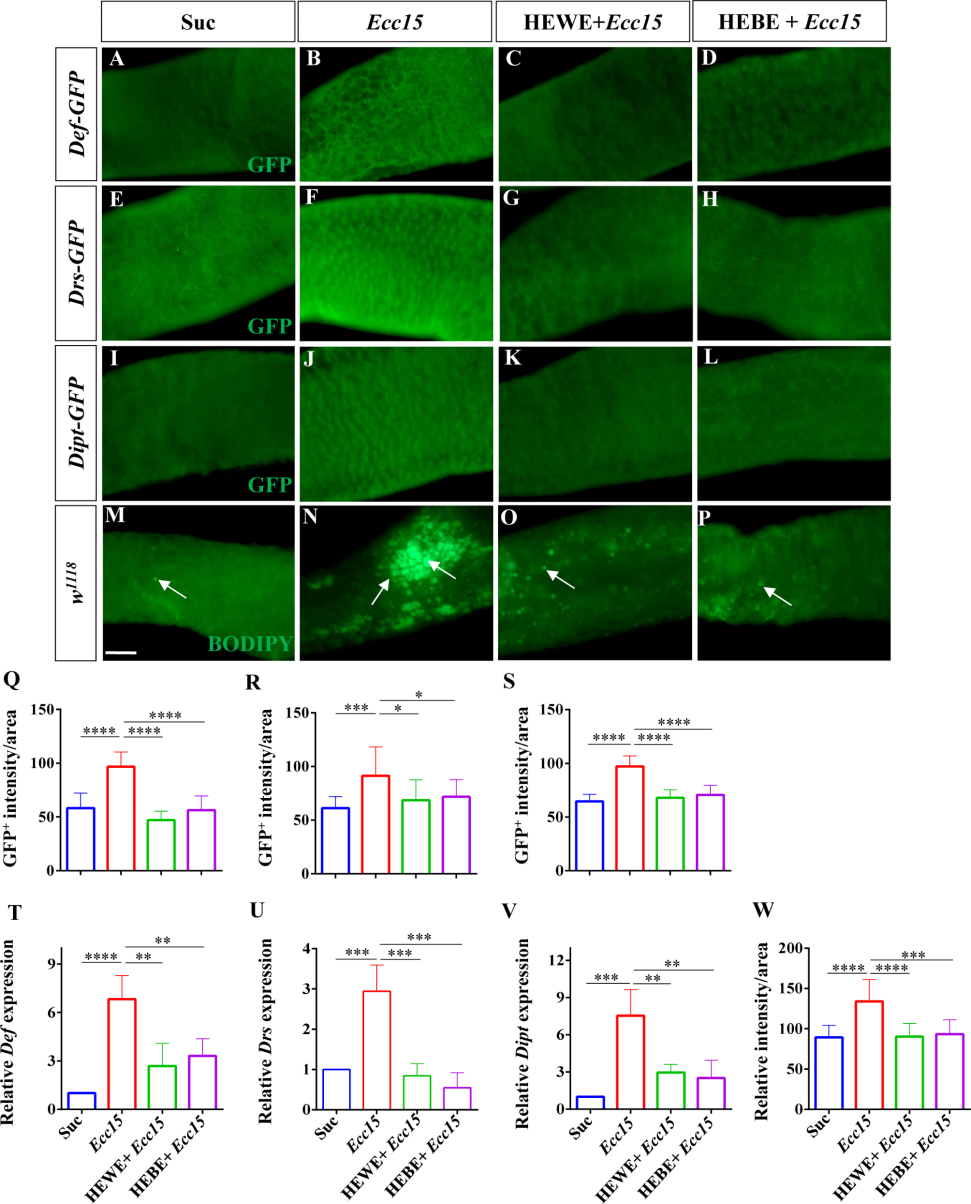
Both HEWE and HEBE can reduce excessive AMP levels and steatosis during *Ecc15* infection. (**A**-**L**) Using anti-GFP antibodies, immunofluorescence images of the posterior midgut were obtained. The transgenic fly lines *Def-GFP* (A-D), *Drs-GFP* (**E**-**H**), and *Dipt-GFP* (**I**-**L**) were infected with *Ecc15* for 16, 16, and 24 h, respectively. Infection with *Ecc15* substantially increased Def, Drs, and Dipt levels. However, supplementation with HEWE or HEBE significantly reduced the increase in AMP levels caused by *Ecc15* infection. (**M**-**P**) Bodipy was used to obtain representative images of midgut lipid droplets (LDs). *Ecc15* infection causes LD accumulation in the midgut, but supplementation with HEWE or HEBE significantly decreases LD accumulation. (**Q**, **R**, **S**) Fluorescence intensities of *Def-GFP* (**Q**), *Drs-GFP* (**R**), and *Dipt-GFP* (**S**) in A-D, E-H, and I-L, respectively (*n* = 12–17). (T-V) After *Ecc15* infection for 6 h, qRT-PCR analysis of Def (**T**), Drs (**U**), and Dipt (**V**) was performed. All mRNA levels were normalized to *rp49* expression. (W) Quantification of the fluorescence intensity of LDs in M-P (*n* = 12–14). Quantification results represent mean ± SEM.*P < 0.05, **P < 0.01,***P < 0.001,****P < 0.0001. Scale bars: 50 μm

Antimicrobial peptides (AMPs) are small, functional proteins with potent antiviral, antibacterial, and antifungal activities [33]. AMPs for host defense against microbial infection in *Drosophila* are produced essentially in adipocytes, but also in gut epithelial cells [34]. According to previous studies, AMP can protect intestinal homeostasis in *Drosophila* by responding to gram-negative bacterial infection [34], but excessive expression of AMP is detrimental to epithelial cells [35–37]. In this study, we examined the expression levels of the reporter genes *defencin* (*Def*)*-GFP*, *drosomycin* (*Drs*)-*GFP*, and *diptericin* (*Dipt*)-*GFP* following infection with the gram-negative bacterium *Ecc15*. The AMP levels were substantially greater in the *Ecc15*-infected group than in the sucrose-fed group (*Def*: *P <* 0.0001, *Drs*: *P <* 0.001, *Dipt*: *P <* 0.0001); however, after the addition of 8 mg/ml HEWE or 1 mg/ml HEBE, the AMP levels decreased to levels comparable to those in the uninfected group (*Def*: HEWE *P <* 0.0001, HEBE *P <* 0.0001; *Drs*: HEWE *P <* 0.05, HEBE *P <* 0.05; *Ditp*: HEWE *P <* 0.0001, HEBE *P <* 0.0001, Fig. 3A-L, Q-S). To further confirm these results, we performed qRT-PCR analysis of AMP gene expression. As anticipated, the *Def*, *Drs*, and *Dipt* transcript levels were 5.8-, 1.9-, and 6.5-fold greater, respectively, in the infected group than in the uninfected group (*Def*: *P <* 0.0001, *Drs*: *P <* 0.001, *Ditp*: *P <* 0.001). In contrast, supplementation with *H. erythrostictum* extracts significantly decreased AMP gene transcription (*Def*: HEWE *P <* 0.01, HEBE *P <* 0.01; *Drs*: HEWE *P <* 0.001, HEBE *P <* 0.001; *Ditp*: HEWE *P <* 0.01, HEBE *P <* 0.01, Fig. 3T-V).

Adult fly bacterial infection induces intestinal steatosis due to lipid accumulation, and intestinal steatosis is an antibacterial immune response marker and modulator [38]. BODIPY staining revealed significant lipid accumulation in response to *Ecc15* infection (*P <* 0.0001); however, supplementation with HEWE or HEBE reduced lipid droplet numbers in the intestine (HEWE: *P <* 0.0001, HEBE: *P <* 0.001, Fig. 3M-P, W). Taken together, these findings showed that *H. erythrostictum* extracts have intestinal-protective effects by regulating excessive AMP levels and lipogenesis in the *Drosophila* gut caused by *Ecc15* infection.

### *H. erythrostictum* extracts eliminate the excessive accumulation of ROS in the intestine

**Fig. 4:**
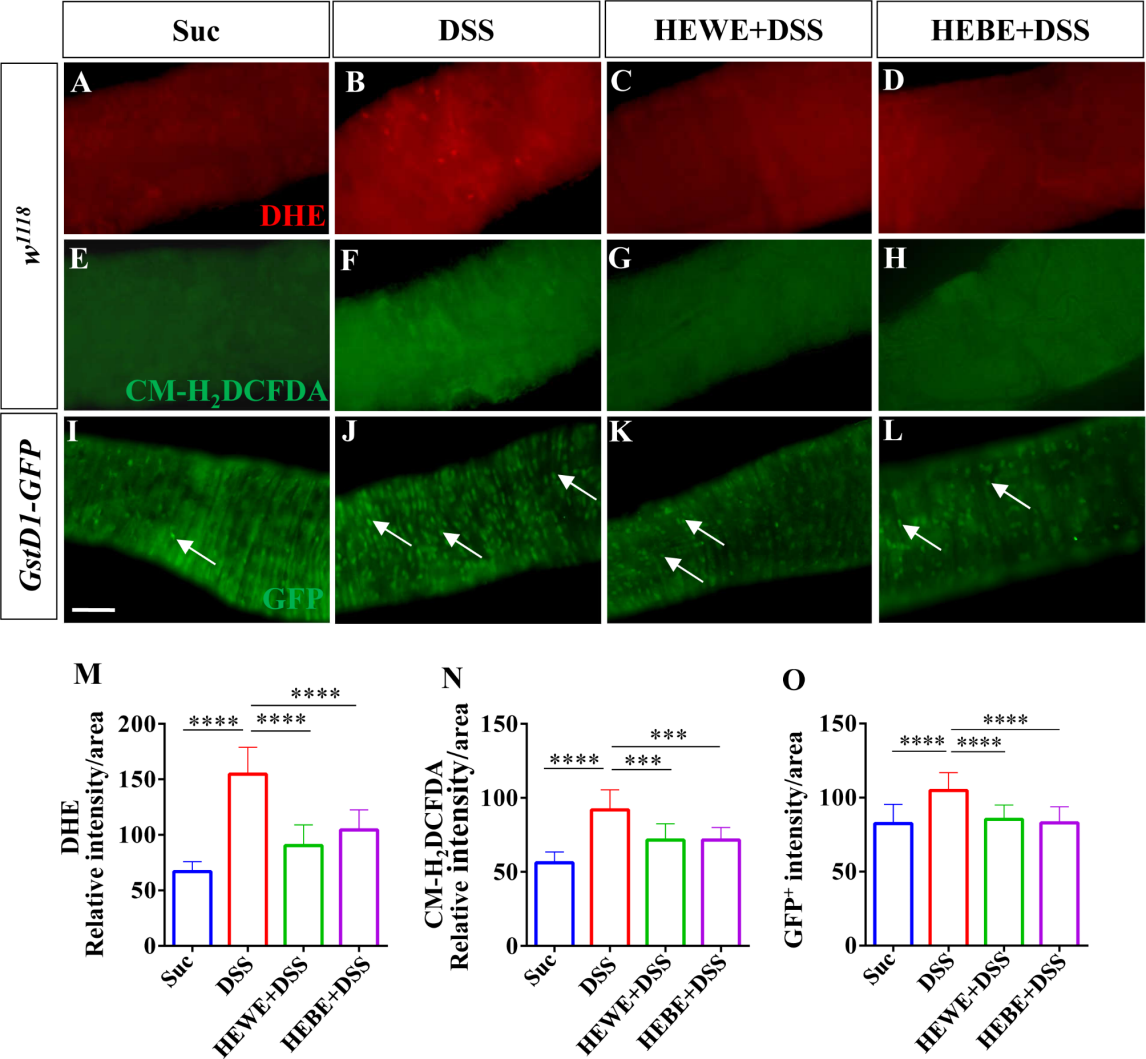
Both HEWE and HEBE can decrease ROS levels in the gut of *Drosophila* fed DSS. (**A**-**H**) ROS levels were monitored with DHE and CM-H_2_CFDA. Wild-type *w*^*1118*^ flies were exposed for 48 h to 6% DSS for DHE staining and for 96 h to 3% DSS for CM-H_2_DCFDA staining. After ingesting DSS, ROS levels in the gut were significantly elevated (**A**, **B**, **E**, **F**). Both HEWE and HEBE ameliorated the DSS-induced increase in ROS levels (**C**, **D**, **G**, **H**). (**I**-**L**) Photographs of the midguts of *gstD1-GFP* transgenic flies. After treatment with 6% DSS for 48 h, the GFP levels increased significantly (**I**, **J**). Supplementation with HEWE or HEBE significantly reduced the expression of gstD1-GFP (**K**, **L**). (**M**) Quantification of DHE intensity in A-D (*n* = 10–16). (**N**) Quantification of CM-H_2_DCFDA intensity in E-H (*n* = 10–16). Quantification of the GFP intensity in I-L (*n* = 12–27). Quantification results represent mean ± SEM.***P < 0.001,****P < 0.0001. Scale bars: 50 μm

In clearing microbes from the gut, reactive oxygen species (ROS), which are highly effective immune effector molecules, exert broad-spectrum microbicidal effects [39]. However, ROS are destructive to intestinal epithelial cells; therefore, their effects must be transient and at low levels [40]. Excessive amounts of ROS cause tissue damage by oxidizing proteins, lipids, and DNA [41]. In the present study, for the first time, the ROS-scavenging activity of *H. erythrostictum* extracts was examined. The levels of ROS in the intestine were measured using dihydroethidium (DHE) and 5-(6)-chloromethyl-2’,7’-dichlorodihydrofluorescein diacetate (CM-H_2_DCFDA). After exposure to DSS, flies had a much stronger fluorescence intensity in the posterior midgut than did those fed sucrose (DHE: *P <* 0.0001, CM-H_2_DCFDA: *P <* 0.0001). However, 8 mg/ml HEWE or 1 mg/ml HEBE supplementation decreased DHE (HEWE: 41.7%, *P <* 0.0001; HEBE: 32.5%, *P <* 0.0001) or CM-H_2_DCFDA (HEWE: 22.2%, *P <* 0.001; HEBE: 22.1%, *P <* 0.0001) fluorescence intensity (Fig. 4A-H, M, N). Next, we evaluated intestinal ROS levels in ROS-sensitive *GstD1-GFP* transgenic flies [42]. In DSS-stimulated flies, the GFP intensity was substantially greater than that in sucrose-fed flies (*P <* 0.0001, Fig. 4I, J, O), corroborating the findings of a previous study indicating that GstD1 activity is increased in differentiated cells in response to stress [43]. As expected, supplementation with HEWE or HEBE significantly decreased GstD1-GFP levels (HEWE: *P <* 0.0001, HEBE: *P <* 0.0001, Fig. 4K, L, O).

Through enzymatic and nonenzymatic antioxidant defenses, living cells have evolved a balanced system to counteract excessive ROS levels [44]. Catalase is one of the oldest known enzymes and has been extensively characterized as a critical defense system against reactive oxygen species and free radicals [45]. This enzyme decomposes H_2_O_2_ into oxygen and water and plays a crucial role in the metabolism of H_2_O_2_ [46]. In addition, superoxide dismutase (SOD) enzymes catalyze the breakdown of superoxide into hydrogen peroxide and water and are central regulators of ROS levels, the first line of defense against free radicals [47, 48]. In a previous study, when excessive ROS were induced with paraquat, ISC proliferation and an increase in progenitor cells were observed [49]. Therefore, we questioned whether Catalase and Sod2 inhibition would result in an increase in progenitor cells. The GFP signal from*esg-Gal4 UAS-GFP* flies can be used to identify both ISCs and EBs in the intestinal tract of *Drosophila.* When *Sod2* or *Catalase* was knocked down in ISCs/EBs using the *esg > GFP* driver, we showed that the numbers of progenitors and mitotic cells were significantly greater than those in*esg > GFP*/+ flies (*Sod2* RNAi: esg^+^ cells *P <* 0.001, PH3^+^ cells *P <* 0.0001; *Catalase* RNAi: esg^+^ cells *P <* 0.0001, PH3^+^ cells *P <* 0.0001, Figure S1A, D, G, J, M-P). However, supplementation with *H. erythrostictum* extracts reduced the numbers of esg^+^ (HEWE: 27.6%, *P <* 0.0001, 41.4%, *P <* 0.001; HEBE: 33.4%, *P <* 0.001, 41.1%, *P <* 0.0001) and PH3^+^ (HEWE: 49.6%, *P <* 0.01, 50.4%, *P <* 0.0001; HEBE, 36.6%, *P <* 0.01, 55.9%, *P <* 0.01) cells compared to those in cells with low levels of antioxidant enzyme-induced increases in cell numbers (Figure S1B, C, E, F, H, I, K, L, M-P). These results indicated that both HEWE and HEBE possess ROS-scavenging activity, which, by reducing ROS accumulation, can reduce progenitor overproliferation and differentiation.

### *H. erythrostictum* extracts inhibit the proliferation and differentiation of stem cells induced by DSS

**Fig. 5:**
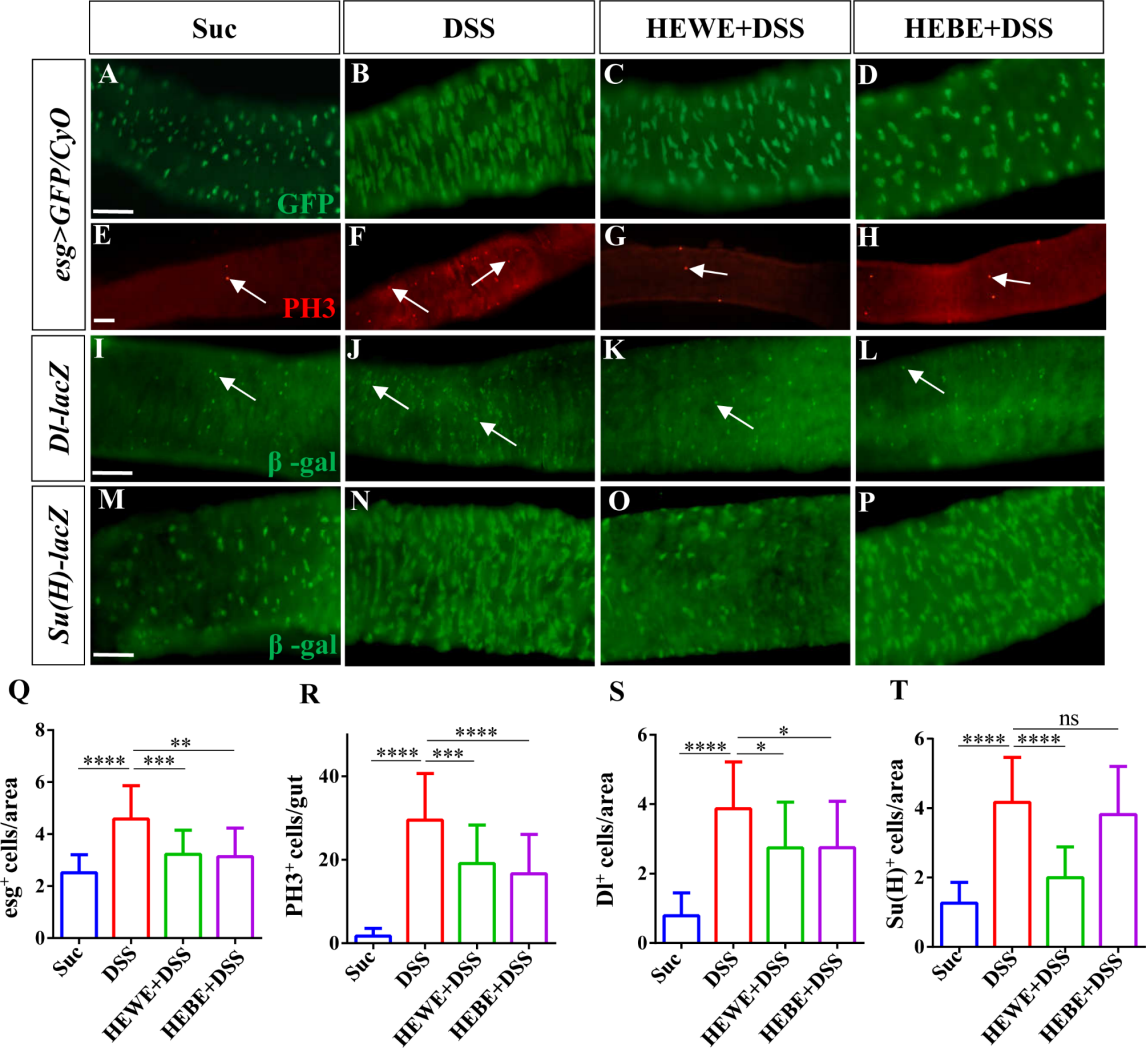
Both HEWE and HEBE prevent excessive proliferation and differentiation of ISCs induced by DSS. (**A**-**P**) Representative images of the posterior midguts of *esg > GFP/Cyo*(ISC/EB marker, **A**-**H**), *Dl-lacZ* (ISC marker, **I**-**L**) and *Su(H)-lacZ* (EB marker, **M**-**P**) transgenic flies. Guts were stained using anti-GFP (green), anti-PH3 (red), and anti-β-gal antibodies (green). After treatment with 3% DSS for 72 h, the numbers of esg^+^ cells, PH3^+^ cells, ISCs, and EBs significantly increased. Both HEWE and HEBE supplementation significantly reduced the increases in all types of targeted cells. However, HEBE did not reduce the increase in EBs. (**Q**-**T**) Quantification of the numbers of esg^+^ cells in **A**-**D** (*n* = 13–30), the numbers of PH3^+^ cells in **E**-**H** (*n* = 25–32), the numbers of ISCs in **I**-**L** (*n* = 12–14), and the numbers of EBs in M-P (*n* = 12–14). Quantification results represent mean ± SEM. ns*P* > 0.05, *P < 0.05, **P < 0.01,***P < 0.001,****P < 0.0001. Scale bars: 50 μm

To preserve the epithelial barrier, microbial or chemical damage to epithelial surfaces activates a number of signal transduction pathways that accelerate progenitor proliferation and differentiation [40]. In a previous experiment, DSS-exposed *Drosophila* intestinal epithelial cells died, and intestinal integrity was compromised. Therefore, we examined whether *H. erythrostictum* extracts could maintain intestinal epithelial homeostasis to protect intestinal integrity. In the midgut epithelium of the sucrose-fed group, modest and dispersed subpopulations of esg^+^ cells were observed (Fig. 5A). Many large and concentrated esg^+^ cells were observed after 72 h of feeding with 3% DSS, and the number of progenitor cells increased by more than 82% (*P <* 0.0001, Fig. 5B, Q). Compared to those in DSS-fed group, supplementation with 8 mg/ml HEWE or 1 mg/ml HEBE significantly reduced the numbers of esg^+^ cells by 29.6% (*P <* 0.001) and 31.6% (*P <* 0.01), respectively (Fig. 5C, D, Q). To further confirm the presence of mitotic stem cells, we analyzed cell proliferation with an anti-phospho-histone H3 (anti-PH3) antibody. After DSS stimulation, the number of PH3^+^ cells in the entire midgut significantly increased (*P <* 0.0001); however, supplementation with HEWE or HEBE inhibited the number of PH3^+^ cells by 35.35% (*P <* 0.001) and 43.6% (*P <* 0.0001), respectively (Fig. 5E-H, R).

Next, we utilized transgenic flies expressing the reporter genes *Delta-lacZ* (an ISC-specific marker) and *Su(H)GBE-lacZ* (an EB-specific marker) to analyze the numbers of ISCs and EBs, respectively. As expected, the numbers of Dl^+^ and Su(H)^+^ cells increased substantially after three days of treatment with 3% DSS (Dl^+^ cells: *P <* 0.0001, Su(H)^+^ cells: *P <* 0.0001). However, both the Dl^+^ and Su(H)^+^ cell numbers decreased by 29.2% (*P <* 0.05) and 52.0% (*P <* 0.0001), respectively, in the HEWE-supplemented group, while only the Dl^+^ cell number decreased by 28.9% (*P <* 0.05) in the HEBE-supplemented group (Fig. 5I-P, S, T). Our results demonstrated that *H. erythrostictum* extracts have a protective effect against DSS-induced abnormal proliferation and differentiation of progenitors.

### *H. erythrostictum* extracts modulate progenitor overproliferation and differentiation by inhibiting the ROS-associated JNK signaling pathway

**Fig. 6:**
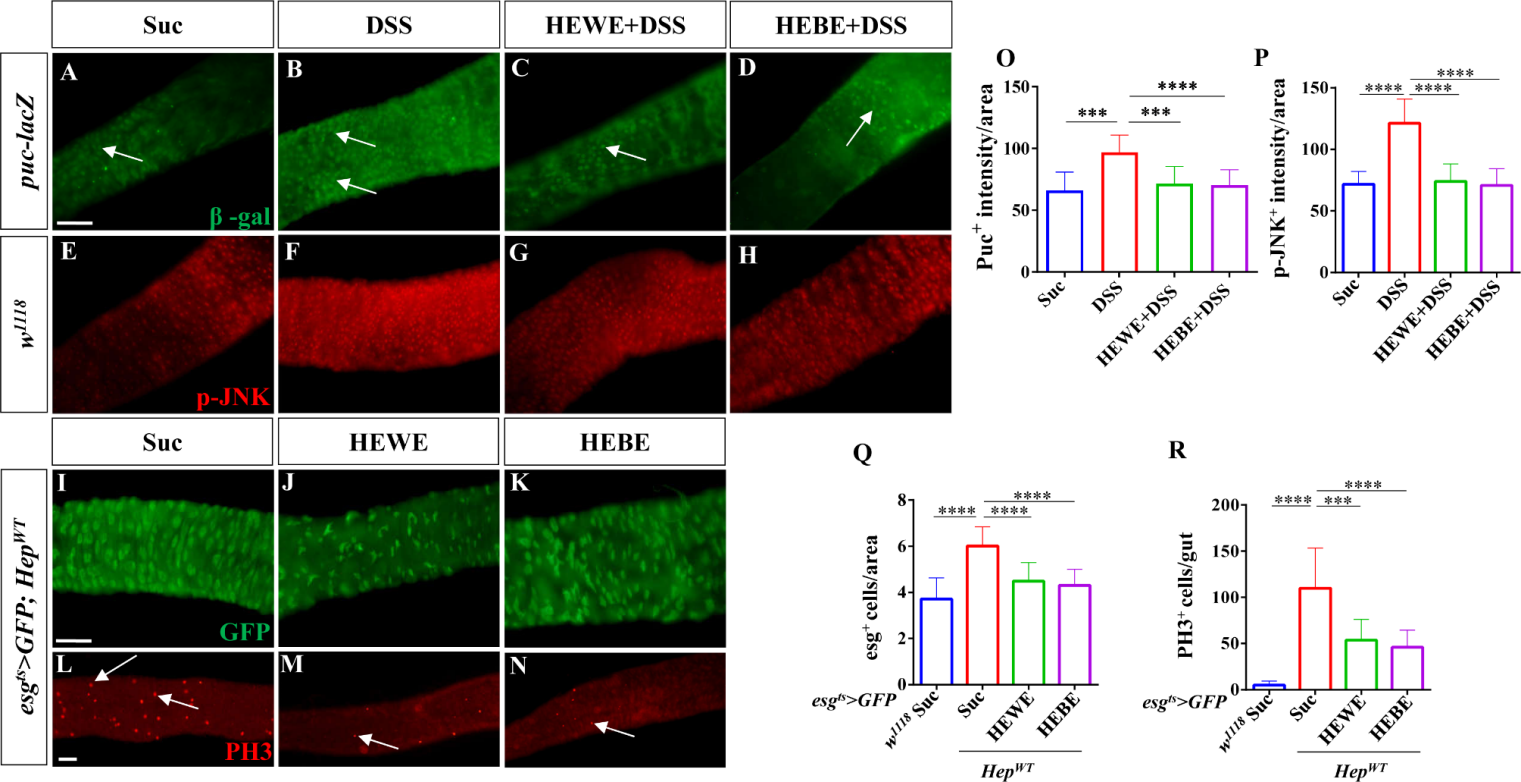
Both HEWE and HEBE inhibited the activation of the JNK pathway by DSS. (**A**-**H**) Representative images of the posterior midguts of *puc-lacZ* (**A**-**D**) and *w*^*1118*^ (**E**-**H**) flies. Guts were stained with anti-β-gal antibodies (green) and anti-p-JNK antibodies (red). After treatment with 3% DSS for 72 h, the number of β-gal^+^ cells and the p-JNK level increased. Both HEWE and HEBE supplementation effectively decreased the increase in the number of β-gal^+^ cells and the level of p-JNK. (**I**-**N**) Representative images of the posterior midgut of *esg*^*ts*^*> GFP; Hep*^*WT*^ transgenic flies stained with anti-GFP (green, **I**-**K**) and anti-PH3 (red, **L**-**N**) antibodies. In flies supplemented with HEWE or HEBE, the numbers of esg^+^ and PH3^+^ cells were significantly lower than those in the sucrose group. (**O**-**R**) Quantification of the numbers of β-gal^+^cells in **A**-**D** (*n* = 12–15), p-JNK intensities in E-H (*n* = 12–13), the numbers of esg^+^ cells in **I**-**K**, and the numbers of PH3^+^ cells in L-N (*n* = 12–16).Quantification results represent mean ± SEM. ***P < 0.001, ****P < 0.0001. Scale bars: 50 μm

ROS-induced cellular damage and mortality, as well as bacterial or chemical ingestion, can activate JNK stress signaling pathways in ECs [50]. Activation of these pathways leads to the nuclear translocation and activation of the transcription factor AP1 (downstream of the JNK pathway), which induces cytokine and growth factor expression. Activation of JNK signaling pathways was observed by monitoring target gene expression. Using *puc-lacZ*flies, we first measured the expression level of a reporter of JNK signaling. After 3 days of treatment with 3% DSS, the number of lacZ^+^ cells significantly increased compared to that in the sucrose-fed group (*P <* 0.0001). However, this increase was completely reversed by supplementation with 8 mg/ml HEWE or 1 mg/ml HEBE (HEWE: *P <* 0.0001, HEBE: *P <* 0.001, Fig. 6A-D, O). An anti-p-JNK antibody was subsequently used to assess JNK pathway activation. *H. erythrostictum* extracts significantly attenuated increase in the fluorescence intensity of p-JNK (HEWE: *P <* 0.0001, HEBE: *P <* 0.0001, Fig. 6E-H, P).To confirm the inhibitory effect of *H. erythrostictum* extracts on the JNK signaling pathway, we specifically overexpressed the JNK kinase hemipterous (Hep) in progenitor cells and counted the numbers of progenitor and mitotic cells. As a controls, the *esg*^*ts*^*> GFP* transgene was crossed with wild-type *w*^*1118*^. In *esg*^*ts*^*> GFP*; *Hep*^*WT*^ transgenic flies, the numbers of esg^+^ and PH3^+^ cells dramatically increased (*P <* 0.0001, *P <* 0.0001); however, after feeding with 8 mg/ml HEWE or 1 mg/ml HEBE, the numbers of progenitor and mitotic cells decreased by more than 25.4% (HEWE: *P <* 0.0001, HEBE: *P <* 0.0001) and 51.1% (HEWE: *P <* 0.001, HEBE: *P <* 0.0001), respectively (Fig. 6I-N, Q, R). These findings demonstrated that *H. erythrostictum* extracts can inhibit excessive progenitor proliferation and differentiation by decreasing JNK pathway activity.

### *H. erythrostictum*extracts regulate intestinal homeostasis by inhibiting the JAK-STAT pathway

**Fig. 7:**
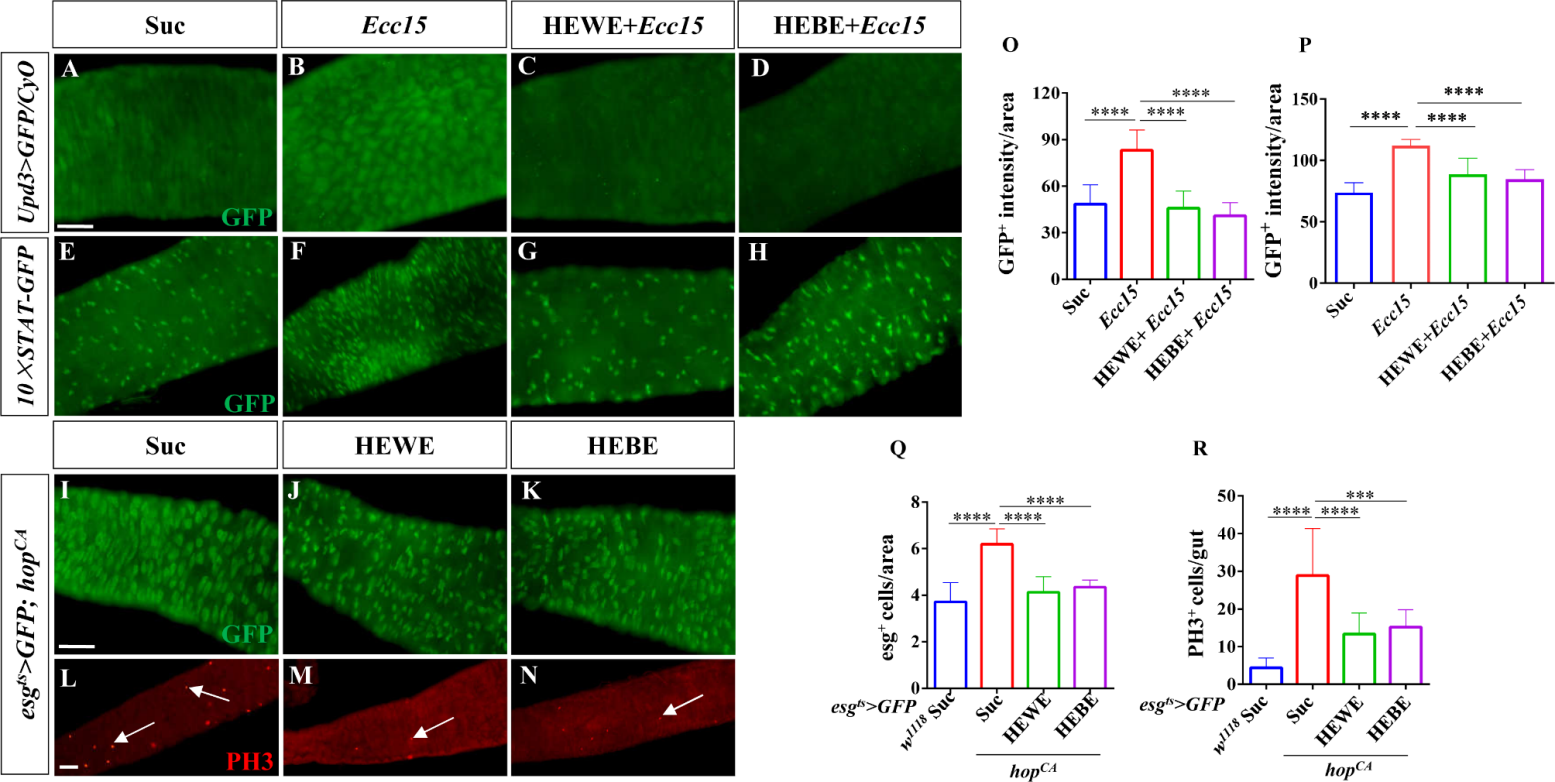
Both HEWE and HEBE inhibited the activation of the JAK–STAT pathway by*Ecc15*. (**A**-**H**) Representative images of the posterior midguts of *upd3 > GFP/CyO* (**A**-**D**) and *10×STAT-GFP* (**E**-**H**) flies. Guts were stained with anti-GFP antibodies (green). After infection with *Ecc15* for 16 h, the intensity of GFP and the number of GFP^+^ cells increased. Both HEWE and HEBE supplementation effectively reduced the increase in the intensity of GFP and the number of GFP^+^ cells. (**I**-**N**) Representative images of the posterior midgut of *esg*^*ts*^*> GFP*; *hop*^*CA*^ transgenic flies. Guts were stained with anti-GFP (green, **I**-**K**) and anti-PH3 (red, **L**-**N**) antibodies. In flies supplemented with HEWE or HEBE, the numbers of esg^+^ and PH3^+^ cells were significantly lower than those in the sucrose group. (**O**-**R**) Quantification of GFP intensities in **A**-**D** (*n* = 11–15), GFP^+^ cell numbers in **E**-**H**, the numbers of esg^+^ cells in **I**-**K**, and the numbers of PH3^+^ cells in L-N (12–19) (*n* = 13–15). Quantification results represent mean ± SEM. ***P < 0.001, ****P < 0.0001. Scale bars: 50 μm

Increased JNK activity in ECs is sufficient to promote ISC proliferation by stimulating the expression of the cytokines Upd2 and Upd3. These cytokines promote regeneration by stimulating JAK/STAT signaling in ISCs to promote their proliferation and in EBs to promote their differentiation [40, 51]. Upd3 is one of the main cytokines that acts as a signal to elicit antibacterial and reparative host responses during oral infection with entomopathogenic bacteria such as *Ecc15* [20, 51]. Therefore, we analyzed Upd3 expression in *upd3 > GFP/CyO* flies. As expected, after oral infection with *Ecc15*, GFP levels increased by more than 71.6% (*P <* 0.0001); however, after supplementation with HEWE or HEBE, GFP signals decreased almost to control levels (HEWE: *P <* 0.0001, HEBE: *P <* 0.0001, Fig. 7A-D, O). Next, the activity of the JAK/STAT pathway was evaluated using the *10XSTAT-GFP* reporter, a common JAK/STAT signaling reporter containing 10 Stat92E-binding sites derived from the suppressor of cytokine signaling at 36E (Soc-s36E) gene [52]. As expected, supplementation with 8 mg/ml HEWE or 1 mg/ml HEBE reduced the number of GFP^+^ cells induced by *Ecc15* (HEWE: *P <* 0.0001, HEBE: *P <* 0.0001, Fig. 7E-H, P).

We overexpressed hopscotch (hop) of *Drosophila* JAK kinase in ISCs/EBs using the *esg*^*ts*^*> GFP* driver to confirm the inhibitory effect of *H. erythrostictum* extracts on the JAK/STAT signaling pathway. The numbers of progenitor and mitotic cells increased by approximately 1.7- and 5.6-fold, respectively (esg^+^ cells: *P <* 0.0001, PH3^+^ cells: *P <* 0.0001). However, supplementation with HEWE or HEBE significantly suppressed the JAK/STAT pathway-induced proliferation and differentiation of cells. The numbers of progenitor and mitotic cells drastically decreased, with the progenitor number resembling that in the sucrose group (esg^+^ cells: HEWE *P <* 0.0001, HEBE *P <* 0.0001; PH3^+^ cells: HEWE *P <* 0.001, HEBE *P <* 0.0001, Fig. 7I-N, Q, R). In conclusion, these findings demonstrated that *H. erythrostictum* extracts inhibit the JAK-STAT pathway to maintain intestinal homeostasis.

### *H. erythrostictum* extracts inhibit intestinal injury induced by DSS via the EGFR pathway

**Fig. 8:**
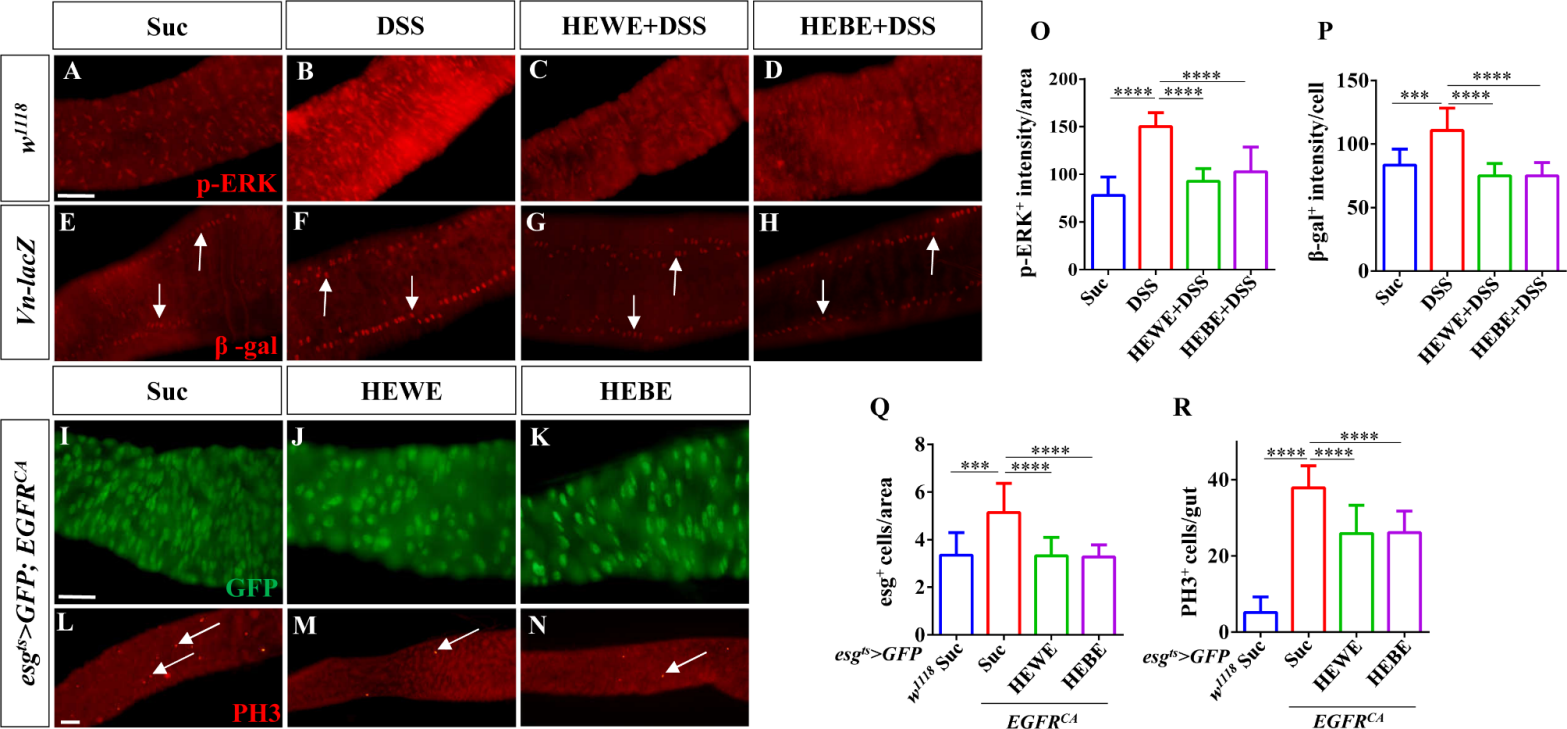
Both HEWE and HEBE inhibited the activation of theEGFR pathway by DSS. (**A**-**H**) Representative images of the posterior midguts of *w*^*1118*^ (**A**-**D**) and *Vn-lacZ* (**E**-**H**) flies. Guts were stained with anti-p-ERK (red) and anti-β-gal antibodies (red). After treatment with 3% DSS for 72 h, the levels of p-ERK and β-gal increased. Both HEWE and HEBE supplementation effectively decreased the elevated p-ERK and β-gal levels. (**I**-**N**) Representative images of the posterior midgut of *esg*^*ts*^*> GFP*; *EGFR*^*CA*^ transgenic flies. Guts were stained with anti-GFP (green, **I**-**K**) and anti-PH3 (red, **L**-**N**) antibodies. In flies supplemented with HEWE or HEBE, the numbers of esg^+^ and PH3^+^ cells were significantly those the sucrose group.(**O**-**R**) Quantification of p-ERK intensities in A-D (*n* = 13–15), β-gal intensities in E-H (*n* = 11–13), the numbers of esg^+^ cells in I-K, and the numbers of PH3^+^ cells in L-N (12–19). Quantification results represent mean ± SEM. ***P < 0.001,****P < 0.0001. Scale bars: 50 μm

The EGFR/Ras/MAPK pathway is crucial for ISC proliferation, and JNK and JAK/STAT signaling induce ISC proliferation by activating EGFR signaling [7, 16]. Therefore, we examined whether *H. erythrostictum* extracts could protect against intestinal injury induced by DSS via the EGFR pathway. We initially stained the midgut with an anti-p-Erk antibody that recognizes the activated form of MAPK [53]. After 3 days of stimulation with 3% DSS, the p-Erk intensity in the midgut increased by approximately 92.5% compared to that in the sucrose-fed group (*P <* 0.0001); however, supplementation with 8 mg/ml HEWE or 1 mg/ml HEBE significantly decreased the p-Erk intensity (*P <* 0.0001, *P <* 0.0001, Fig. 8A-D, O). Three EGFR ligands, Vein, Spitz, and Keren, are expressed in the intestine of *Drosophila*. Vein is expressed in the visceral mesoderm (VM) surrounding the intestinal epithelium in a stable state and is the most essential ligand for stimulating ISC division [7, 54, 55]. The expression level of the EGFR signaling pathway ligand was subsequently detected using the *Vein* (*Vn*)*-lacZ* reporter. The fluorescence intensity of Vn-lacZ^+^ cells in the midgut was significantly greater in the DSS-fed group than in the sucrose-fed group (*P <* 0.001). Supplementation with 8 mg/ml HEWE or 1 mg/ml HEBE significantly decreased Vein expression in response to DSS stimulation (HEWE: *P <* 0.0001, HEBE:*P <* 0.0001, Fig. 8E-H, P).

The inhibitory effect of *H. erythrostictum* extracts on the activation of the EGFR pathway was also evaluated. Compared to those in the control group, the numbers of progenitor and mitotic cells in the EGFR pathway-activated flies increased by approximately 1.5- and 7.3-fold, respectively (esg^+^ cells: *P <* 0.001, PH3^+^ cells: *P <* 0.0001). The numbers of esg^+^ and PH3^+^ cells decreased substantially after supplementation with 8 mg/ml HEWE or 1 mg/ml HEBE (esg^+^ cells: HEWE *P <* 0.0001, HEBE *P <* 0.0001; PH3^+^ cells: HEWE *P <* 0.0001, HEBE *P <* 0.0001, Fig. 8I-N, Q, R). In response to DSS-induced intestinal injury, these findings demonstrated that *H. erythrostictum* extracts can protect against intestinal epithelial homeostasis by inhibiting the EGFR signaling pathway.

### Effects of *H. Erythrostictum* extracts on the composition and abundance of the gastrointestinal microbiota

**Fig. 9:**
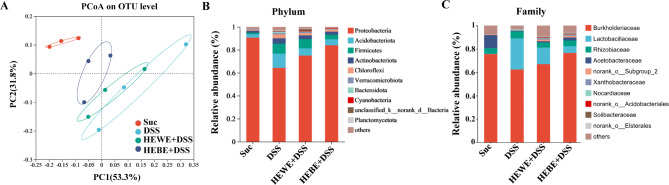
Effects of HEWE and HEBE on the gut microbiome structure in DSS-fed flies. (**A**) Beta diversity analysis of the gut microbiota in each group using the PCoA method based on Bray curtis values. (**B**) Relative abundance at the phylum level for each group (*n* = 3). (**C**) Relative abundance at the family level for each group (*n* = 3)

The gut microbiota may play a role in modulating ISC proliferation following tissue damage and boosting the host immune system [8]. Therefore, we used 16 S rDNA sequencing to determine whether the anti-inflammatory effects of *H. erythrostictum* extracts on DSS-induced inflammation are associated with alterations in the intestinal microbial structure. Alpha diversity analysis was performed to calculate the species diversity of samples using indices such as Ace, Shannon, and Simpson indices, as depicted in Figure S2A-C. There were no significant differences among the four distinct diet groups. A Venn diagram was generated to illustrate the unique and overlapping gut operational taxonomical units (OTUs) among the different groups. Following supplementation with HEWE or HEBE, the number of OTUs induced by DSS in the intestine decreased (Figure S2D). Beta diversity analysis was also performed to investigate the similarities and differences in community composition between samples from distinct groups. Principal coordinate analysis (PCoA) based on Bray curtis values was performed to visualize the similarity between microbiota compositions. As shown in Fig. 9A, the Suc, DSS, HEWE + DSS, and HEBE + DSS groups had distinct gut microbial compositions, whereas the HEWE + DSS and HEBE + DSS groups had similar gut microbial compositions, indicating that *H. erythrostictum* extracts modulated gut microbiota dysregulation in DSS-fed flies.

**Fig. 10:**
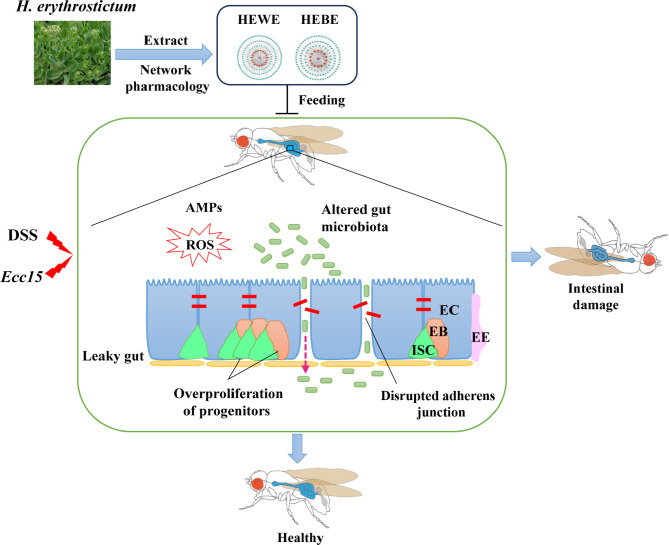
Mechanism of action of HEWE and HEBE against intestinal injury induced by DSS and *Ecc15*

Figure 9B shows the relative abundance levels of the top 10 bacterial taxa in the intestines of the four distinct groups at the phylum level. *Proteobacteria*, *Acidobacteriota*, and *Firmicutes* were demonstrated to be the predominant phyla in eachgroup. Compared to those in the sucrose-fed group, the *Acidobacteriota* (12.4%) and *Firmicutes* (8.6%) in the DSS-fed group increased, and the relative abundance of *Proteobacteria* decreased (64.3%). Nevertheless, the HEWE and HEBE groups exhibited relative decreases in *Acidobacteriota* (6.3% and 5.2%) and *Firmicutes* (8.1% and 4.0%) and relative increases in *Proteobacteria* (74.9% and 83.9%).

Subsequently, we analyzed the gut microbiota composition of each group at the family level.As shown in Fig. 9C, *Burkholderiaceae*, *Lactobacillaceae*, *Rhizobiaceae*, and *Acetobacteraceae* were the dominant family found in the gut of each group, respectively.Compared to those in the sucrose-fed group, the *Lactobacillaceae* (26.6%) and *Rhizobiaceae* (5.9%) in the DSS-fed group increased, and the relative abundance of *Burkholderiaceae* (62.4%) and *Acetobacteraceae* (1.05%) decreased. Nevertheless, the HEWE and HEBE groups exhibited relative decreases in *Lactobacillaceae* (14.0% and 5.8%) and *Rhizobiaceae* (4.5% and 4.8%) and relative increases in *Burkholderiaceae* (67.0% and 76.6%) and *Acetobacteraceae* (1.08% and 2.7%).Shifts in relative abundance of various bacterial phyla and families indicated that DSS induced substantial alterations in the gut microbiota, as demonstrated by our findings. Moreover, our findings demonstrated the effect of HEWE or HEBE supplementation on the intestinal microbiota.

Changes in the proportions of *Proteobacteria*, *Acidobacteriota*, and *Firmicutes*at phylum level and *Burkholderiaceae*, *Lactobacillaceae*, *Rhizobiaceae*, and *Acetobacteraceae* at family level suggested that *H. erythrostictum* extracts may selectively stimulate the growth of particular beneficial bacteria while inhibiting the growth of harmful bacteria. These results suggested that *H. erythrostictum* extracts could be a viable therapeutic agent for restoring the gut microbiota balance and improving gut homeostasis.

### Analysis of active compounds and potential targets of *H. erythrostictum*extracts

To identify the bioactive compounds present in HEWE and HEBE, we conducted metabolite profiling of the *H. erythrostictum* extracts. Widely targeted metabolomics analysis identified 633 (HEWE) and 512 (HEBE) compounds in *H. erythrostictum* extracts. Of these compounds, 42 (HEWE) and 21 (HEBE) compounds with relative contents of more than 0.1%, high gastrointestinal absorption and excellent drug likeness were selected. Based on these results, SwissADME predicted 492 and 347 targets in HEWE and HEBE, respectively. Various databases, including OMIM (253 targets), CTD (164 targets), DisGeNet (731 targets), GeneCards (948 targets), and TTD (62 targets), were consulted to obtain the IBD targets, and the database intersections are displayed in Figure S3A. The intersection of compound targets and IBD targets revealed 166 and 120 hub targets, respectively (Figure S3B, C). These results demonstrated that *H. erythrostictum* extracts contain potential IBD-treating compounds. To predict the anti-inflammatory bioactive compounds of HEWE and HEBE against IBD, a network of herb-compound targets was constructed using Cytoscape software. (Figure S3D, E). The results indicated that 19 compounds in HEWE and 14 components in HEBE could be candidate bioactive compounds for IBD treatment (Tables S1, S2). These results demonstrated that *H. erythrostictum* extracts can be used to treat IBD effectively via multiple targets and compounds.

## Discussion

Fruit flies are commonly used as powerful models for drug testing. Xiu et al. emphasized that *Drosophila* is a suitable model for high-throughput screening of drugs derived from natural products [56]. The *Drosophila* intestinal epithelium is an important defense barrier that prevents infection and invasion of the microbiota or toxic substances; moreover, the fly intestine is structurally, genetically, and functionally similar to the mammalian intestine, making it an ideal model for studying the mechanisms of intestinal disorders [57, 58]. Thus, numerous researchers are currently examining natural molecules and herbal extracts with distinct protective and therapeutic effects on intestinal inflammation [56].

The protective effects of *H. erythrostictum* water extract (HEWE) and *H. erythrostictum* butanol extract (HEBE) on preventing intestinal injury caused by DSS stimulation or *Ecc15* infection have been described in this study (Fig. 10). DSS or SDS was administered with various concentrations of HEWE or HEBE, and 8 mg/ml HEWE and 1 mg/ml HEBE resulted in greater survival rates. Our previous studies revealed that ingestion of SDS or DSS in fruit flies can lead to damage of intestinal barrier integrity and epithelial cell death, significantly reducing the survival rate of the flies [59–61]. Supplementation with HEWE and HEBE was able to reverse intestinal epithelial cell death and resulted in disorganized cell localization and disruption of intestinal barrier integrity and acid‒base homeostasis in *Drosophila* intestinal disorders induced by DSS. In addition, we did not observe a difference in food intake between the control group and those fed HEWE or HEBE.

We observed that infection with the gram-negative bacterium *Ecc15* significantly increased intestinal AMP levels. It has been well established that AMPs serve as effective antimicrobial agents against a variety of pathogens. However, increasing evidence suggests that excessive AMPs may have other physiological effects that may be harmful to host tissues, such as modulating the microbiome and being involved in nervous system activity and aging [62]. Our data demonstrated that *H. erythrostictum* extracts can reduce excessive AMP levels induced by *Ecc15*. In addition, Harsh et al. described the function of lipid droplets (LDs) in the context of host–pathogen interactions and immunity. Systemic infection with *Photorhabdus* bacteria causes gastrointestinal steatosis characterized by LD accumulation, which is related to the activation of immune signaling pathways. Moreover, intestinal steatosis is an indicator and regulator of the antibacterial immune response [38]. Our results revealed substantial accumulation of intestinal LDs upon infection with *Ecc15*, which was consistent with previous results of infection with gram-negative *Photorhabdus* bacteria [38]. Nonetheless, supplementation with HEWE or HEBE substantially decreased LDs accumulation, indicating that *H. erythrostictum* extracts can modulate the immune response by inhibiting *Ecc15*-induced excessive lipogenesis.

ROS are groups of active molecules derived from molecular oxygen and are typical examples of double-edged swords [63]. Appropriate levels of ROS serve as signaling molecules that modulate particular signaling pathways to maintain normal cell function and survival [64]. However, excessive ROS can injure tissues through the oxidation of proteins, lipids, and DNA [42]. Moreover, in *Drosophila*, elevated ROS levels can cause intestinal injury by increasing the proliferation of ISCs [65]. This study revealed that HEWE and HEBE inhibited DSS-induced accumulation of ROS in the midgut. In addition, HEWE and HEBE inhibited *UAS-Cat RNAi-* and *UAS-Sod2 RNAi*-induced proliferation and mitosis of ISC progenitors. These findings demonstrated that *H. erythrostictum* extracts possess ROS-scavenging activity and can inhibit the excessive proliferation and differentiation of progenitors caused by elevated ROS levels.

ISC differentiation and proliferation are required to maintain intestinal homeostasis in the presence of toxic compounds or bacterial infection; however, excessive proliferation and differentiation can lead to a variety of diseases, including inflammatory bowel disease and cancer. By inhibiting the JNK, JAK-STAT, and EGFR pathways, HEWE or HEBE supplementation contributed to the maintenance of ISC homeostasis in the context of DSS ingestion and *Ecc15* infection. In addition, 16 S rDNA sequencing of the gut microbiota revealed that the amelioration of DSS-induced inflammation by *H. erythrostictum* extracts was associated with alterations in the structure of the gut microbiota. After supplementation with HEWE or HEBE, the proportions of *Proteobacteria*, *Acidobacteriota*, and *Firmicutes* changed significantly at the phylum level.

We performed metabolite profiling and network pharmacology analysis of *H. erythrostictum* extracts to investigate the essential components and mechanisms of HEWE and HEBE in the treatment of IBD. Based on our network pharmacology analysis, 19 major components of HEWE and 14 major components of HEBE were linked to IBD hub targets (Tables S1, S2). Previous studies have shown that *Ecc15* infection causes intestinal injury, increases intestinal progenitor numbers, and leads to morphological changes [16]. However, there are no studies describing whether *Ecc15* can induce IBD, though it may contribute to inflammatory triggers or exacerbate IBD. However, the specific functions and mechanisms of these active ingredients in treating inflammatory intestinal injury have not been fully elucidated, necessitating further clarification through the use of multiple IBD models.

## Conclusion

These findings demonstrate that *H. erythrostictum* extracts have a potential effect in alleviating intestinal inflammation caused by DSS ingestion or *Ecc15* infection in a *Drosophila* model. Therefore, *H. erythrostictum* extracts have distinct protective effects against intestinal damage and may be novel, effective treatments for gut inflammation.

## Electronic supplementary material

Below is the link to the electronic supplementary material.

Supplementary Material 1

Supplementary Material 2

Supplementary Material 3

Supplementary Material 4

## Data Availability

Data from this study are available from the corresponding author upon reasonable request.

## Abbreviations

H.erythrostictum: Hylotelephium erythrostictum (Miq.) H. Ohba
HEWE: Hylotelephium erythrostictumwater extract
HEBE: Hylotelephium erythrostictumbutanol extract
IBD: Inflammatory bowel disease
ROS: Reactive oxygen species
DSS: Dextran sodium sulfate
SDS: Sodium dodecyl sulfate
Ecc15: Erwinia carotovoracarotovora 15
LD: Lipid droplet
AMP: Antimicrobial peptide
ISC: Intestinal stem cell
EB: Enteroblast
EC: Absorbent enterocyte
EE: Enteroendocrine cell
PBS: Phosphate buffered saline
PBST: Triton X-100 in PBS
PFA: Paraformaldehyde
BPB: Bromophenol blue sodium
7-AAD: 7-Aminoactinomycin D
Def: Defencin
Drs: Drosomycin
Dipt: Diptericin
OTUs: Operational taxonomical units
CCR: Copper cell region
