# Supplementary material for: Extracts ofHylotelephiumerythrostictum (miq.) H. Ohba ameliorate intestinal injury by scavenging ROS and inhibiting multiple signaling pathways in Drosophila
Source: BMC Complement Med Ther. 2024 Nov 14;24:397. doi: 10.1186/s12906-024-04686-w (PMC11566468; doi:10.1186/s12906-024-04686-w)
Supplement: Supplementary file 1 — Supplementary Material 1 [file 12906_2024_4686_MOESM1_ESM.pdf]

Extracts of *Hylotelephium erythrostictum* (Miq.) H. Ohba ameliorate intestinal injury by scavenging ROS and inhibiting multiple signaling pathways in *Drosophila*

Hyonil Kim[1,2], Xinyu Yi[1], Hongmei Xue[3], Guanhua Yue[5], Jiahua Zhu[5], Tongju Eh[1,2], Sihong Wang[4]*, Li Hua Jin[1]*

Supplemental information

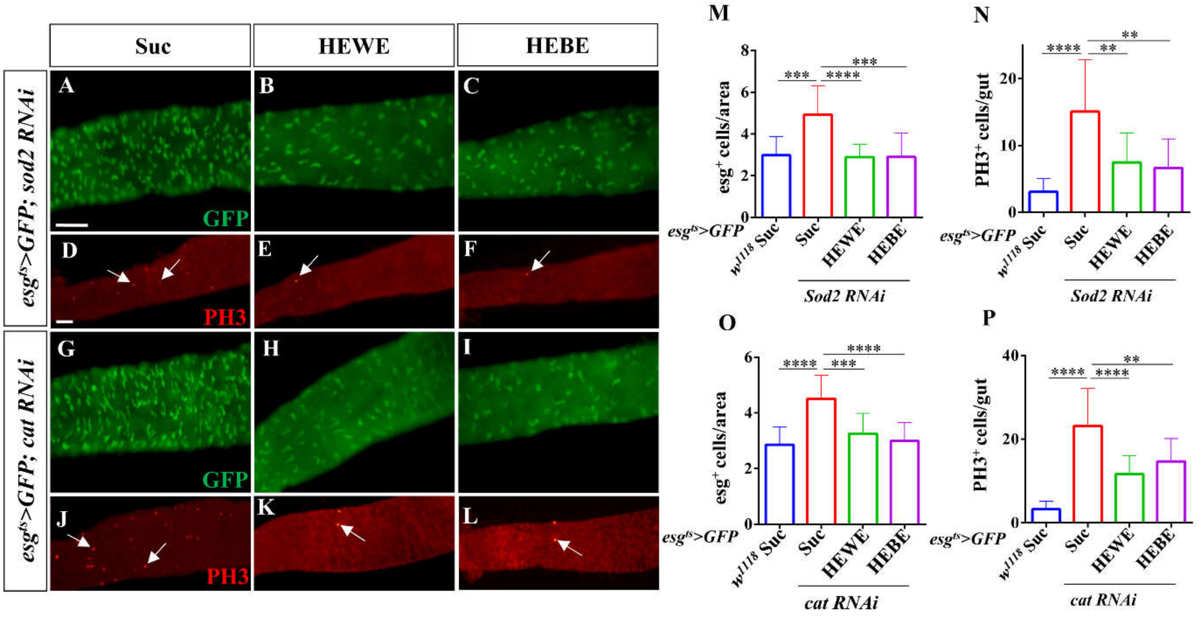

Figure S1. Both HEWE and HEBE can reduce progenitor proliferation and differentiation caused by the suppression of antioxidant enzymes.

(A-L) Representative images of the midguts of *esg^{ts}>GFP; sod2 RNAi* (A-F) and *esg^{ts}>GFP; cat RNAi* (G-L) transgenic flies at 29 °C for 7 days and stained with anti-GFP (A-C, G-I) and anti-PH3 (D-F, J-L) antibodies. The *esg-Gal4/+* flies were used as controls. The numbers of esg^{+} and PH3^{+} cells were significantly greater in the *esg^{ts}>GFP; sod2 RNAi* and *esg^{ts}>GFP;*

cat RNAi groups than in the control group. After 4 days of supplementation with HEWE or HEBE, the numbers of esg$^+$ and PH3$^+$ cells decreased significantly. (M-P) Quantification of esg$^+$ cells (M, O) in the posterior midgut and PH3$^+$ cells (N, P) in the whole intestine from A-L (n=12-18). Scale bars: 50 μm.

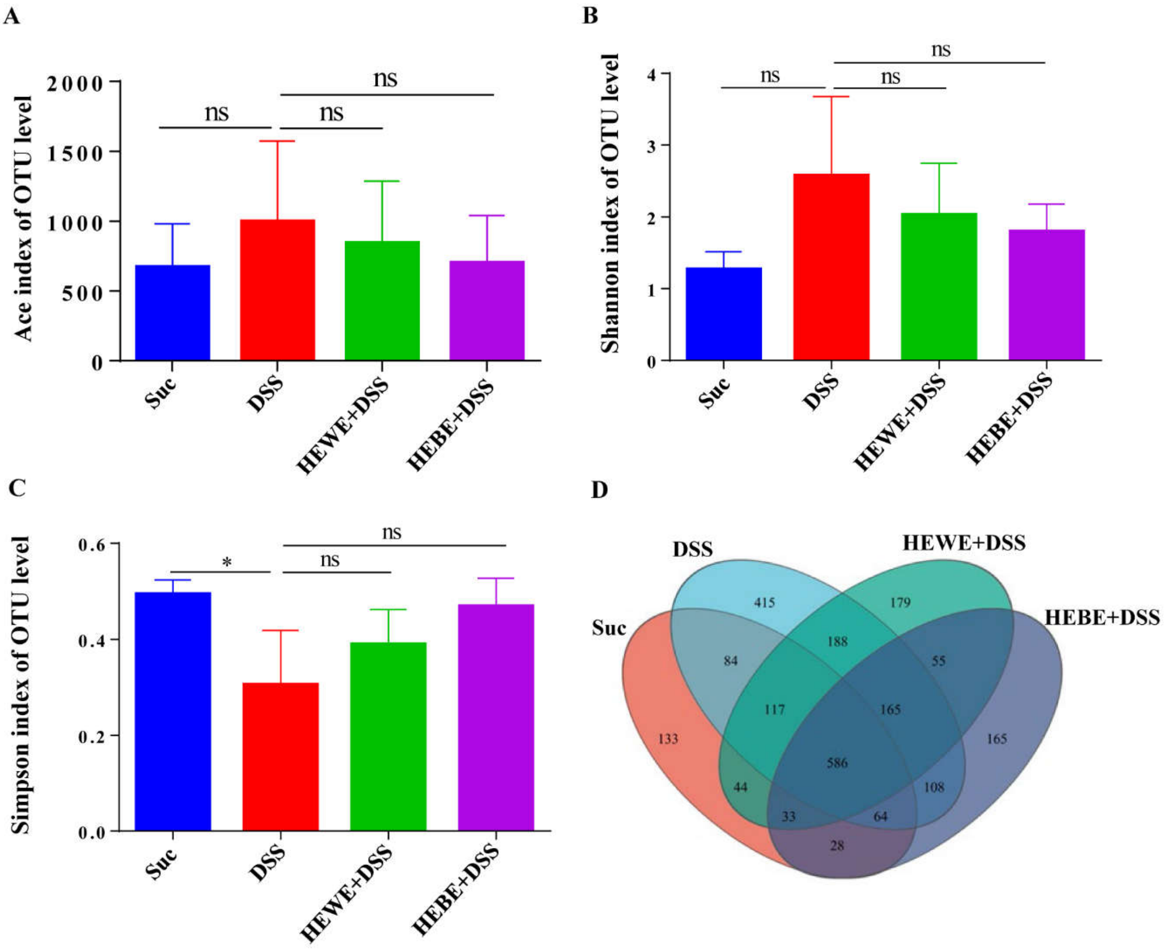

Figure S2. Alpha diversity and Venn diagram of gut microbiota.

(A-C) Alpha diversity bar charts (Ace, Shannon, and Simpson indices) for each group (n=3). (D) Venn diagram showing OTU overlaps between each group.

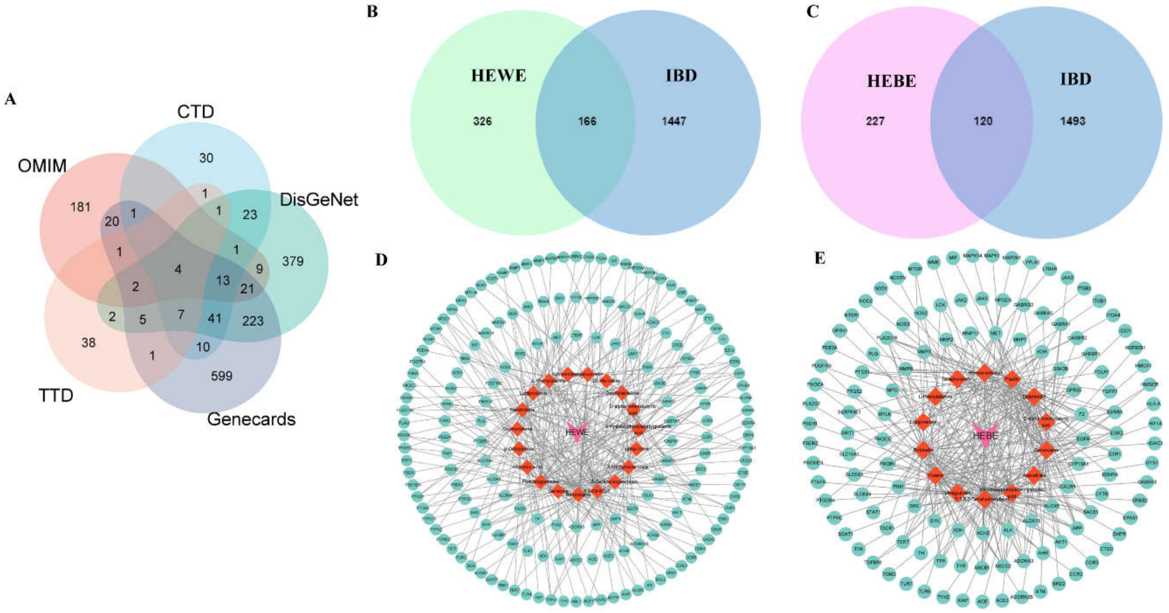

Figure S3. Key compounds and targets of HEWE and HEBE for the treatment of IBD.

(A) Venn diagrams of IBD targets from 5 databases. (B) Intersection of identified targets of HEWE and IBD. (C) Intersection of identified targets of HEBE and IBD. (D, E) Network of herb-compound–target interactions. The red diamonds are the compounds of HEWE and HEBE, and the green circles around them are the targets.

Table S1. Bioactive compounds of HEWE.

No	Compound name	PubChem CID	Class	GI absorption	DL
1	DL-Norvaline	824	Amino acid and derivatives	High	Yes
2	Deethylatrazine	22563	Triazines	High	Yes
3	D-alpha-Aminobutyric acid	439691	Carboxylic acids and derivatives	High	Yes
4	4-Hydroxyphenylacetylglutamic acid	440731	Glutamic acid and derivatives	High	Yes
5	alpha-Irone	5371002	Prenol lipids	High	Yes
6	8-Methylnonenoate	5365959	Fatty Acyls	High	Yes
7	8-Geranyloxypsoralen	5317564	Coumarins	High	Yes
8	8,9-DiHETrE	3246873	Fatty Acyls	High	Yes
9	Benzocaine	2337	Benzene and substituted derivatives	High	Yes
10	Sarracine	5281746	Alkaloids	High	Yes
11	Piperlonguminine	5320621	Alkaloids	High	Yes
12	Phloretic acid	10394	Phenols	High	Yes
13	p-Octopamine	4581	Phenols	High	Yes
14	Oxymorphone	5284604	Phenanthrenes and derivatives	High	Yes
15	Nandrolone	9904	Steroids and steroid derivatives	High	Yes
16	Lubiprostone	157920	Fatty Acyls	High	Yes
17	L-Phenylalanine	6140	Amino acid and derivatives	High	Yes
18	Kynurenic acid	3845	Alkaloids	High	Yes
19	Isosakuranetin	160481	Flavonoids	High	Yes

Table S2. Bioactive compounds of HEBE.

No	Compound name	PubChem CID	Class	GI absorption	DL
1	Homoeriodictyol	73635	Flavonoids	High	Yes
2	Fisetin,	5281614	Flavonoids	High	Yes
3	Delphinidin	68245	Flavonoids	High	Yes
4	D-alpha-Aminobutyric acid	439691	Carboxylic acids and derivatives	High	Yes
5	Benzocaine	2337	Benzene and substituted derivatives	High	Yes
6	Acevaltrate	65717	Iridoids	High	Yes
7	4-Hydroxyphenylacetylglutamic acid	440731	Glutamic acid and derivatives	High	Yes
8	2',3,5,7-Tetrahydroxyflavon	5281610	Flavonoids	High	Yes
9	Veraguensin	443026	Lignans	High	Yes
10	Tricetin	5281701	Flavonoids	High	Yes
11	Robinetin	5281692	Flavonoids	High	Yes
12	Lubiprostone	157920	Fatty Acyls	High	Yes
13	L-Phenylalanine	6140	Amino acid and derivatives	High	Yes
14	Isosakuranetin	160481	Flavonoids	High	Yes

There are no Gels and Blots images in this study. Therefore, we did not provide the full uncropped Gels and Blots images.
