# Supplementary figures and images for: Extracts ofHylotelephiumerythrostictum (miq.) H. Ohba ameliorate intestinal injury by scavenging ROS and inhibiting multiple signaling pathways in Drosophila
Source: BMC Complement Med Ther. 2024 Nov 14;24:397. doi: 10.1186/s12906-024-04686-w (PMC11566468; doi:10.1186/s12906-024-04686-w)

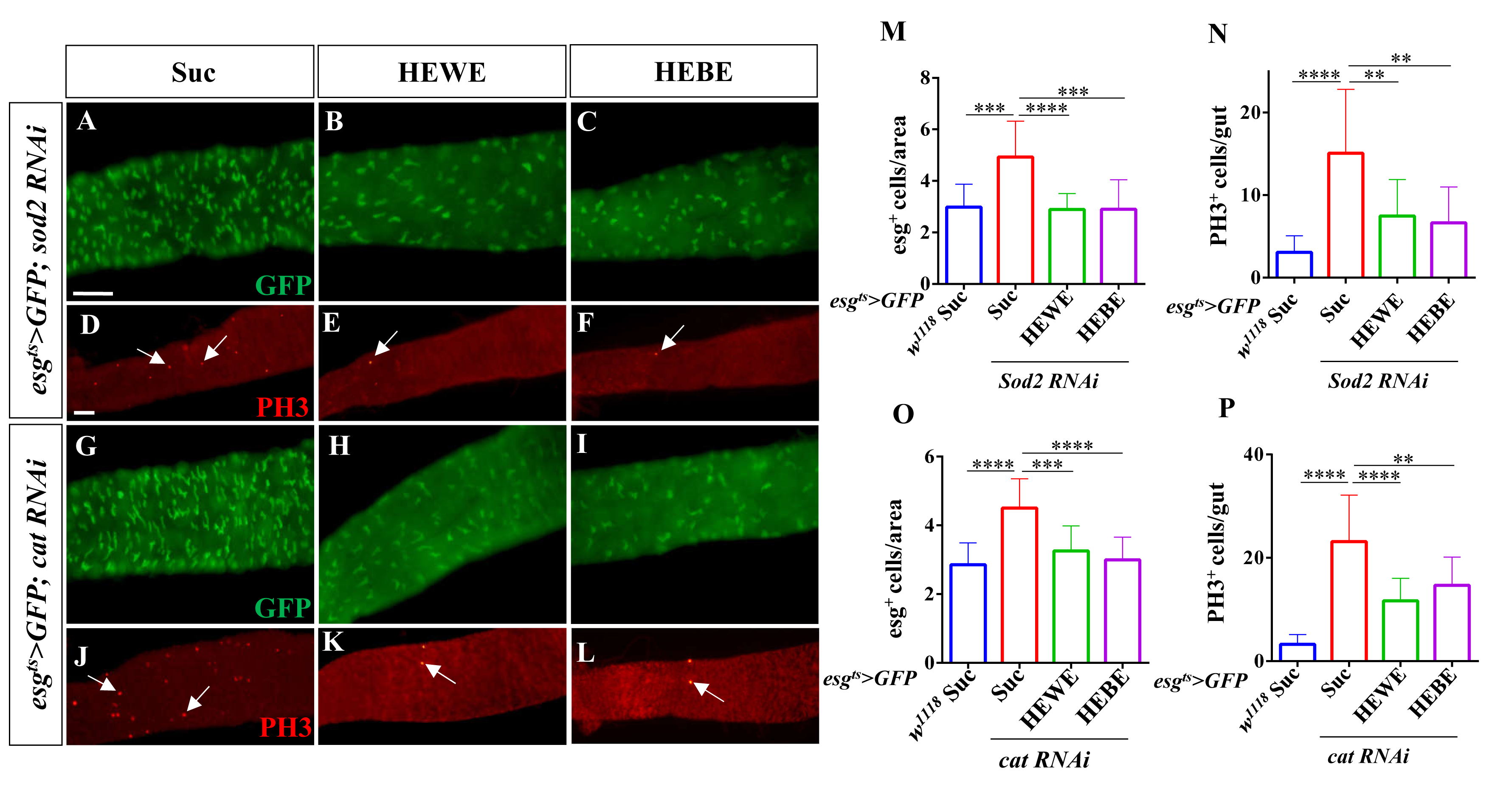

Supplement: Supplementary file 2 — Supplementary Material 2 [file 12906_2024_4686_MOESM2_ESM.tif]

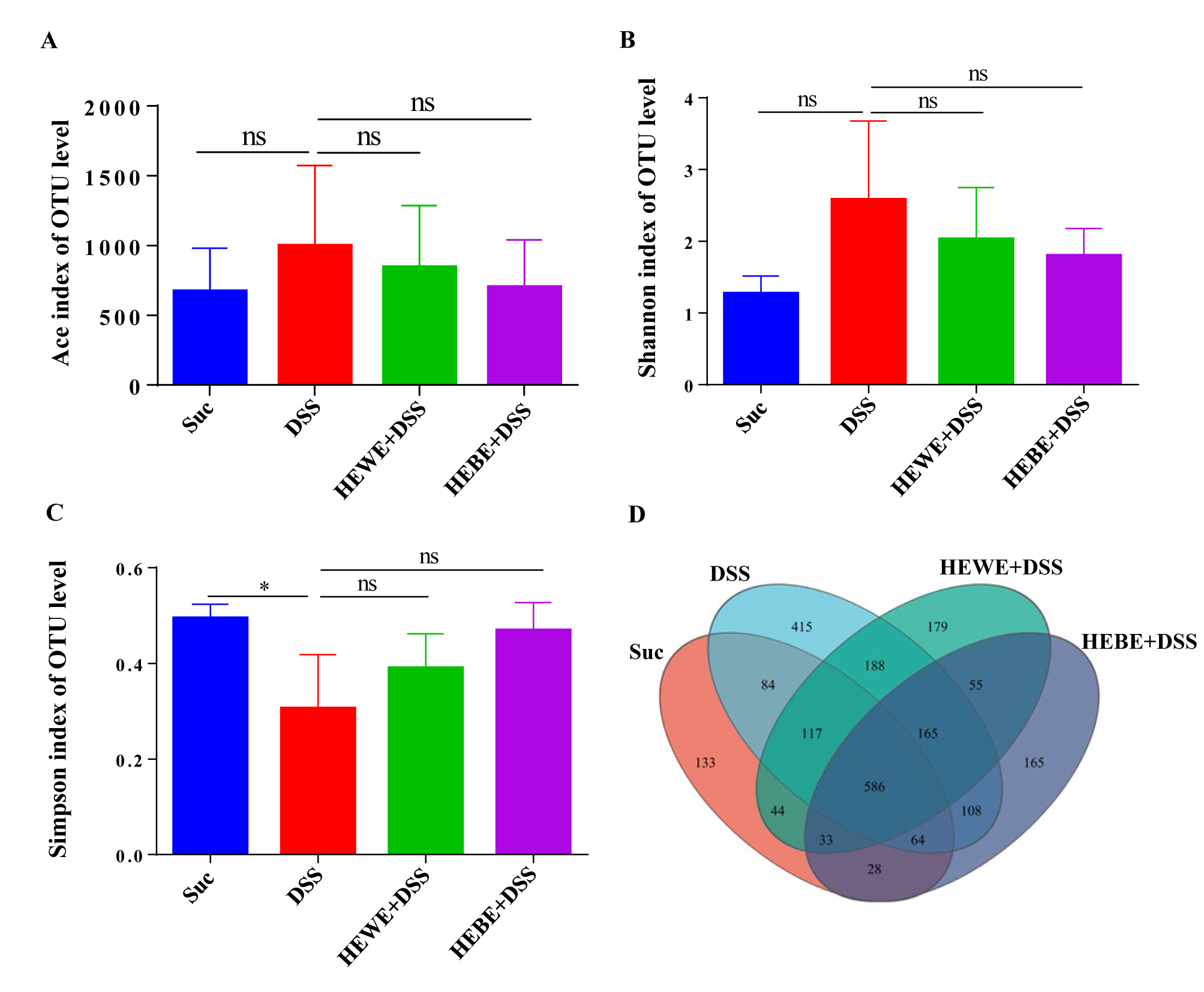

Supplement: Supplementary file 3 — Supplementary Material 3 [file 12906_2024_4686_MOESM3_ESM.tif]

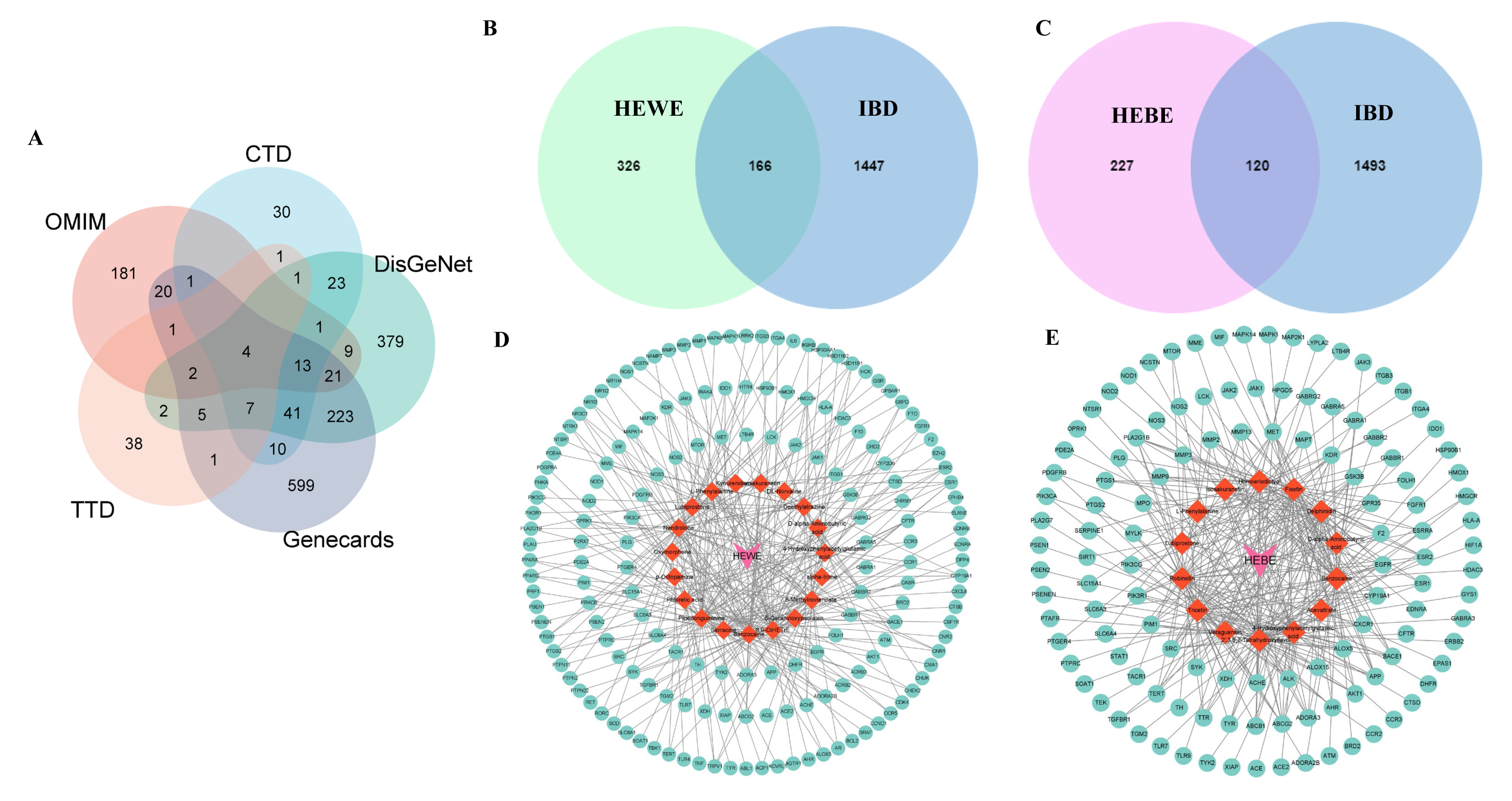

Supplement: Supplementary file 4 — Supplementary Material 4 [file 12906_2024_4686_MOESM4_ESM.tif]
